# A supervised learning framework for chromatin loop detection in genome-wide contact maps

**DOI:** 10.1038/s41467-020-17239-9

**Published:** 2020-07-09

**Authors:** Tarik J. Salameh, Xiaotao Wang, Fan Song, Bo Zhang, Sage M. Wright, Chachrit Khunsriraksakul, Yijun Ruan, Feng Yue

**Affiliations:** 1 Bioinformatics and Genomics Program, The Pennsylvania State University, University Park, State College, PA 16802 USA; 2 Department of Biochemistry and Molecular Genetics, Northwestern University Feinberg School of Medicine, Chicago, IL 60611 USA; 3 The Jackson Laboratory for Genomic Medicine, Farmington, CT USA; 4 Department of Genetics and Genome Sciences, University of Connecticut Health Center, Farmington, CT USA; 5 Robert H. Lurie Comprehensive Cancer Center of Northwestern University, Chicago, IL USA

**Keywords:** Gene regulatory networks, Genome informatics, Chromatin structure

## Abstract

Accurately predicting chromatin loops from genome-wide interaction matrices such as Hi-C data is critical to deepening our understanding of proper gene regulation. Current approaches are mainly focused on searching for statistically enriched dots on a genome-wide map. However, given the availability of orthogonal data types such as ChIA-PET, HiChIP, Capture Hi-C, and high-throughput imaging, a supervised learning approach could facilitate the discovery of a comprehensive set of chromatin interactions. Here, we present Peakachu, a Random Forest classification framework that predicts chromatin loops from genome-wide contact maps. We compare Peakachu with current enrichment-based approaches, and find that Peakachu identifies a unique set of short-range interactions. We show that our models perform well in different platforms, across different sequencing depths, and across different species. We apply this framework to predict chromatin loops in 56 Hi-C datasets, and release the results at the 3D Genome Browser.

## Abstract (web summary)

Predicting chromatin loops from genome-wide interaction matrices such as Hi-C data provides insight into gene regulation events. Here, the authors present Peakachu, a Random Forest classification framework that predicts chromatin loops from genome-wide contact maps, and apply it to systematically predict chromatin loops in 56 Hi-C datasets, with results available at the 3D Genome Browser.

## Introduction

The proper gene regulatory programs of mammalian cells are largely influenced by the 3D conformation of chromosomes^1^. At kilobase to megabase scales, gene promoters are often connected to their distal regulatory elements, such as enhancers, through chromatin loops; rewiring of such loops has been implicated in developmental diseases and tumorigenesis^2,3^. It has been shown that chromatin loops are mediated by architectural proteins CTCF and cohesin via a loop extrusion model, in which CTCF binds to a specific and non-palindromic motif in a convergent orientation at two sites, acting as loop anchors^4,5^.

A growing number of experiments have been used to detect chromatin loops. Hi-C^6^, a high-throughput derivative of Chromosome Conformation Capture (3C)^7^, quantifies contacts between all possible pairs of genomic loci using a proximity-ligation procedure. With an improved experimental protocol and deep sequencing, in situ Hi-C^8,9^ makes it possible to detect loops at kilobase resolution. By introducing micrococcal nuclease for chromatin fragmentation instead of restriction enzymes, Micro-C^10^ further enables nucleosome-resolution analysis of chromatin interactions. Proximity-ligation techniques also include ChIA-PET^11^, PLAC-Seq^12^, and HiChIP^13^, which detect loops bound to target proteins through chromatin immunoprecipitation steps, and include Capture C^14^ and Capture Hi-C^15^, which enrich interactions among a given set of sequences. Recently, several ligation-free techniques emerged to measure different aspects of chromatin organization. Genome Architecture Mapping (GAM)^16^ quantifies chromatin contacts by sequencing DNA from a set of ultrathin nuclear sections at random orientations. Trac-looping^17^ captures multiscale contacts by inserting a transposon linker between interacting regions. DNA SPRITE^18^ follows a split-pool procedure to assign unique barcodes to individual complexes, with read pairs sharing identical barcodes treated similarly to contacts in Hi-C. Besides these biomedical protocols, high-throughput imaging approaches such as STORM^19^ and HiFISH^20^ can directly measure spatial distances at the single-cell level.

As these protocols emerged, investigators accordingly developed computational tools to identify chromatin loops. For Hi-C data: Fit-Hi-C^21^ performs a distance-dependent spline fitting procedure to refine its global background and chooses a binomial distribution as the null model to evaluate contact significance, which can output ~1 million cis-interactions from deeply sequenced reads^22^. HiCCUPS^8,23^ incorporates local background into its model and utilizes the Poisson test with a modified Benjamini–Hochberg adjustment to determine significance, and generally reports thousands of loop interactions. Analysis of ChIA-PET and similar types of data usually starts with peak calling to identify anchor regions for a target protein, but different computational tools may be based on different distributions. For example, the first published ChIA-PET tool^24,25^ adopts a hyper-geometric distribution to filter out noise, while the more recent Mango^26^ software builds a null model by incorporating both the genomic distance and read depth of each anchor. For PLAC-Seq and HiChIP, the recently developed MAPS^27^ filters original interactions against ChIP-Seq peaks of the same protein, and conducts a specific normalization procedure before evaluating significance. For Capture Hi-C, the main data analysis challenges are the asymmetry of interaction matrices, the uneven capture efficiency of baits, and the huge number of tests at single-fragment resolution. To address these problems, CHiCAGO adopts a convolution background model and alleviates multiple testing via a p-value weighting procedure^28^. Alternatively, ChiCMaxima avoids statistical tests by using strategies from the signal processing field to find local maxima and integrates biological replicate information to reduce false-positive rates^29^. We observe that nearly all available tools are based on testing for significant enrichment compared to a local or global background, with specific calculations being quite empirical and difficult to generalize between techniques. It would be intriguing and potentially beneficial to automatically distinguish loop vs non-loop interactions in a data-driven manner, which is a standard supervised learning task.

Machine learning (ML) has been successfully applied in genomics settings, such as predicting microRNA target activities^30^, annotating chromatin states^31,32^, and characterizing functional effects of noncoding variants^33^. In chromatin conformation studies, manifold learning strategies are employed by miniMDS^34^ and GEM^35^ to estimate 3D structures from 2D contact maps. Some investigators have applied ML algorithms to predict 3D interactions from 1D sequence and epigenomic datasets^36,37^. In addition, we recently developed HiCPlus^38^, which can greatly enhance the Hi-C data resolution through a deep convolutional neural network. So far, the potential benefits of ML approaches for loop detection at kilobase scales are relatively unexplored.

**Fig. 1:**
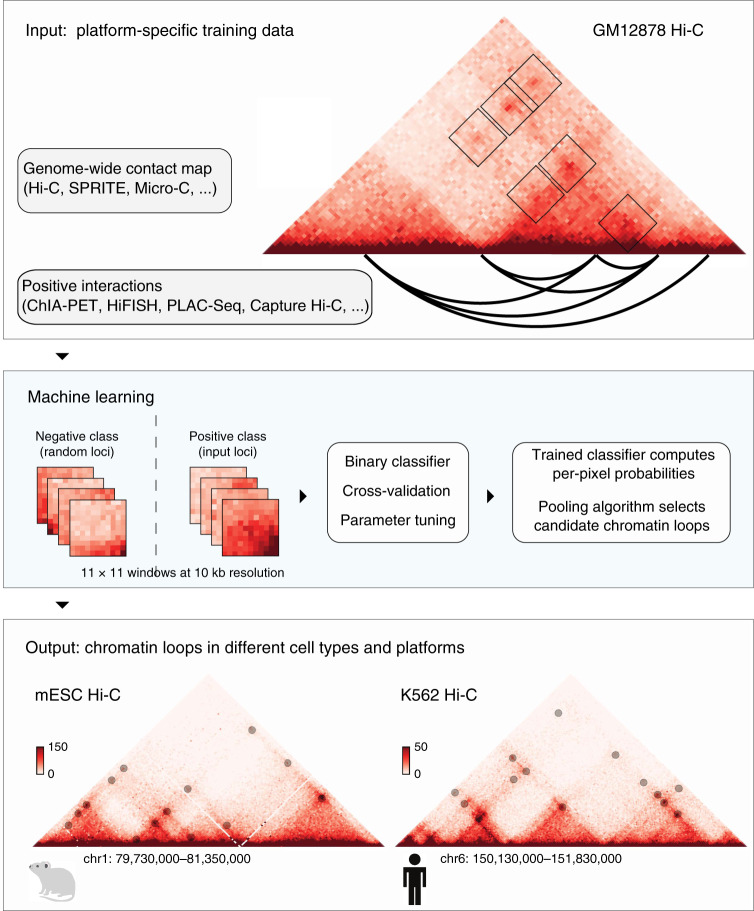
A binary classification framework for loop detection in genome-wide contact data. A contact matrix from Hi-C or similar experiment is decomposed into a training set defined by sub-windows either centered at positive interactions from an orthogonal method (ChIA-PET, PLAC-Seq, HiFISH, Capture Hi-C,…) or random loci of similar genomic distance. Hyperparameter tuning within a 3-fold cross-validation is applied to select a random forest model best able to distinguish the two classes. A trained model can then compute per-pixel probabilities in a different contact map from the same platform, with Hi-C depicted here. A greedy pooling algorithm selects the best-scored contacts from clusters of high-probability pixels.

Here we present Peakachu (Unveil Hi-C Anchors and Peaks) (Fig. 1), a supervised ML framework for detecting chromatin loops from genome-wide interaction maps. Peakachu builds loop-classifying models from defined positive and negative training sets: the positive set could be any list of interactions from either biologically enriched experiments such as ChIA-PET/HiChIP and Capture Hi-C, or a high-throughput imaging experiment such as HiFISH. The negative set is generated from loci randomly sampled from two populations: (1) contacts with genomic distances similar to the positive set, and (2) contacts with larger genomic distances than the positive set. Once the training set is defined, Peakachu applies a hyperparameter search to find the best random forest model separating the two classes, which can be used to detect loops from genome-wide contact maps. We show that the predictions made by Peakachu have high precision and recall rates. Further, we demonstrate Peakachu can detect high-resolution chromatin loops with as few as 30 million intrachromosomal Hi-C reads. With pretrained models, we successfully predict chromatin loops in 56 Hi-C datasets at different sequencing depths and make them available at the 3D Genome Browser (3dgenome.org). Finally, we show Peakachu is a platform-agnostic tool by applying it in two additional genome-wide interaction data types, Micro-C, and DNA SPRITE.

## Results

### Overview of the Peakachu framework

We describe the overall approach by Peakachu in Fig. 1. There are two parts of the input. The first part is a genome-wide interaction matrix, such as Hi-C or Micro-C data. The second part consists of the positive and negative training datasets. Positive training sets are defined by loops identified from orthogonal techniques such as ChIA-PET, PLAC-Seq, Capture Hi-C, or even high-resolution imaging data as they become available. For negative training sets, an equal number of pixels are randomly selected from nonzero values with a distance distribution derived from the positive set. The negative set always contains contacts with the similar genomic distance resembling the positive set, plus a set of long-range contacts resembling noise inherent to contact maps.

The feature vectors of training samples are defined by the surrounding pixels of each sample. Each vector includes the absolute value of each pixel as well as the relative rank of each pixel within the sample. The exact window size is configurable, and we use 11 × 11 windows at 10 kb resolution for all work presented in this text. Once feature vectors are constructed from the positive and negative training sets, Peakachu applies a 3-fold cross-validation loop to select a random forest model (refer to the Methods section for performance comparisons with other machine-learning frameworks) that best separates the two classes. Briefly, the input is randomly separated into three equal parts and multiple models are trained using several combinations of tuning parameters. Each of these models is trained on two parts of the data; one part is used for scoring. The parameter combination achieving the best score is used to fit a final model using the whole training set.

In the prediction stage, similar feature vectors are defined for all nonzero values in a contact map to compute per-pixel probability scores, then a pooling algorithm is applied to eliminate local loop redundancy. A model trained from a given type of contact map, such as Hi-C, can be used to predict loops from other maps of the same platform. As shown in the following sections, a model trained on one cell type can be used to predict loops from Hi-C matrices in other cell types with comparable performance. Detailed description of the framework can be found in the Methods section.

### Orthogonal datasets reveal different sets of chromatin loops

To train and evaluate the performance of Peakachu, we first used the high-resolution Hi-C data from lymphoblastic cell line GM12878^8^, a tier one ENCODE cell line with extensive epigenome data available. There are five types of orthogonal data available in this cell line: CTCF ChIA-PET^24^, RAD21 ChIA-PET^39^, SMC1 HiChIP^13^, H3K27ac HiChIP^40^, and promoter Capture Hi-C^28^ (Supplementary Data 1). First, we observed that each of these enrichment-based assays predicted a unique set of chromatin interactions (Supplementary Fig. 1). Among them, CTCF ChIA-PET identified the highest number of chromatin loops, while H3K27ac HiChIP only identified 6395 loops. 25% (1584 out of the 6395) H3K27ac HiChIP loops were predicted by all five techniques, while 18% (1144/6395) are uniquely predicted in this dataset. Similarly, 32% of the CTCF ChIA-PET loops are unique, while only 7% of them can be recovered by all techniques, potentially due to the fact that the number of loops in CTCF ChIA-PET is much larger than other data types.

**Fig. 2:**
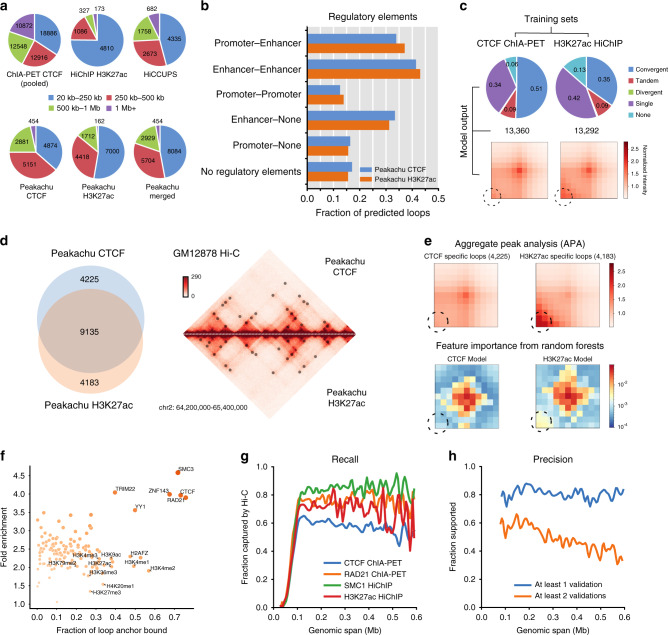
Peakachu framework applied in GM12878 Hi-C. **a** Distance distributions of CTCF ChIA-PET, H3K27ac HiChIP, and HiCCUPS (Hi-C) interactions in GM12878 (top row). Distributions of Peakachu loops predicted from Hi-C after training with CTCF ChIA-PET or H3K27ac HiChIP data, and union of both predictions (bottom row). Interactions in CTCF ChIA-PET were first pooled to remove local redundancy with the same algorithm used by Peakachu. **b** Proportion of predicted loops with different regulatory element combinations at anchor loci. **c** CTCF binding patterns and APA analysis of Peakachu predictions. **d** Overlap of loops predicted by Peakachu models trained with either CTCF ChIA-PET or H3K27ac HiChIP examples, and visualization of interactions predicted from both models. **e** Different features drive the predictions for CTCF and H3K27ac models. APA plots for loops uniquely predicted by CTCF or H3K27ac models (top row). The feature importance metric from random forests showing which pixels drive the classification most strongly (bottom row). **f** Fraction and enrichment of Peakachu loop anchors bound for 133 transcription factors and 10 histone modifications. **g** Fraction of GM12878 interactions in orthogonal experiments recaptured by merged Peakachu loops. **h** Fraction of Peakachu predictions validated by orthogonal experiments. Source data are available in the Source Data file.

More interestingly, we found that these five datasets identified chromatin loops at different genomic distance. For example, 75% (4810/6395) of the H3K27ac HiChIP loops are within 250 kb (Fig. 2a and Supplementary Fig. 2a) and only 8% (500/6395) are over 500 kb. On the contrary, 42% (23,420/55,222) of the pooled CTCF ChIA-PET interactions are long-range (>500 kb) and only 34% are short-range (<250 kb). This is consistent with previous observations that CTCF is more responsible for long-range interactions^41,42^, and that CTCF is a key component of both the loop extrusion model^4,5^ and the formation of TADs^43^. At the same time, H3K27ac is a histone mark for active enhancers and promoters, and therefore it is possible that contacts enriched for H3K27ac are shorter-range interactions between promoters and enhancers, which could be more dynamic than CTCF loops^44^. In the following sections, we first evaluate models trained by both CTCF ChIA-PET and H3K27ac HiChIP and then combine their results to achieve a more comprehensive set of interactions from Hi-C data.

### Peakachu captures known interactions from Hi-C data

We first trained our model in GM12878 Hi-C data with the 92,807 published chromatin interactions from CTCF ChIA-PET in the same cell type (Supplementary Fig. 3). Loops in each chromosome were predicted using models trained in the other 22 chromosomes (Methods). Genome-wide, we identified 13,360 intrachromosomal loops. Aggregate peak analysis (APA) shows that there is an enrichment of Hi-C signals in the predicted loop regions (Fig. 2c). Among the predicted loops, 51% have convergent CTCF binding motifs in binding sites, 34% contain CTCF binding sites at a single anchor, and 9% contain tandem motifs (Fig. 2c). 34% of the predicted loops contained promoters at one anchor and enhancers at another, and 41% contained enhancers at both anchors (Fig. 2b). Seventeen percentage of the predictions contained neither promoters nor enhancers at either anchor.

Next, we trained a model using the 6395 GM12878 H3K27ac HiChIP loops^40^ that contain a higher prevalence of short-range interactions (Supplementary Fig. 3). In total, this model predicted 13,292 loops in the Hi-C matrix. We noticed that 65% of the predictions are the same as from the model trained with CTCF: 65% of which exactly matched 64% of predictions from the CTCF model (8606 of 13,292 and 13,360). When allowing for mismatches of two bins for either anchor, the overlap increases to 69 and 68% (9135 CTCF loops matching 9109 H3K27ac loops) (Fig. 2d). The enrichment for promoters and enhancers is similar but slightly higher than observed from the CTCF ChIA-PET model (Fig. 2b): 37% of the predicted loops are between candidate enhancers and promoters, and ~46% are between enhancers and enhancers.

However, there are differences in the predictions from the two models that vividly reflect the difference in the positive training data. Firstly, we observed a higher percentage of short-range interactions in predictions from the H3K27ac HiChIP model. 53% (7000/13,292) are short-range (<250 kb) while 14% are over 500 kb (Fig. 2a). On the contrary, only 36% (4874/13,360) of predictions by the CTCF model were less than 250 kb, while 25% (3335/13,360) were greater than 500 kb (Fig. 2a). This suggests that H3K27ac HiChIP model identifies more short-range interactions, and CTCF ChIA-PET model is better at identifying long-range loops. Examining the Random Forest feature importance, we found that the most important predictor in a CTCF model is the center pixel, while the H3K27ac model is additionally driven by the lower-left pixels (Fig. 2e). Secondly, we observed a lower CTCF percentage when training with H3K27ac HiChIP, compared with the model trained using CTCF ChIA-PET data (Fig. 2c): 35% of the loops have convergent CTCF binding motifs (vs. 51% in the model trained with CTCF ChIA-PET), 42% contain binding sites at one anchor, and 13% contain no CTCF binding sites. These disparate distributions of CTCF patterns in the prediction are consistent with the patterns in the positive training sets of both models (Supplementary Fig. 2b).

Despite the difference in genomic distance and CTCF motif composition, we observed high validation rates for both prediction sets (Supplementary Fig. 4). Eighty-four percent (11,151 of 13,292) of loops from the H3K27ac HiChIP model and 83% (11,143 of 13,360) of the CTCF ChIA-PET models can be supported by at least one source, while 61% of HiChIP model and 55% (7414 of 13,360) of ChIA-PET model can be supported by at least two sources (Supplementary Fig. 4). Considering predictions unique to either model, we found that 66% (2789 of 4225) of loops from the CTCF model and 68% (2824 of 4183) from the H3K27ac model could be supported by at least one orthogonal source. At least two sources could support 27% (1146 loops) of predictions unique to the CTCF model, and 44% (1827 loops) unique to the H3K27ac model.

Given that both models uniquely predicted valid loops, we decided to use their merged, non-redundant output to report loops from GM12878 and other cell types. This set of predictions from GM12878 Hi-C has high recall and validation rates (Fig. 2g, h) when compared with four validation sets. Nearly 80% of Peakachu-predicted loops can be supported by at least one orthogonal method. We also found that these predicted loops are usually between distal regulatory elements and CTCF binding sites (Supplementary Figs. 5 and 6).

To investigate whether models trained with CTCF ChIA-PET and H3K27ac HiChIP data can also predict loops involving other transcription factors (TFs) and histone modification markers, we computed fold enrichment at loop anchors for 133 TFs and 10 histone modifications from the ENCODE consortium, following a similar approach described in Rao et al. (Methods)^8^. We observed a full range of TFs and histone modifications were enriched in our predicted loops (Fig. 2f and Supplementary Fig. 7), including factors such as YY1^41^, ZNF143^45^, and H3K27me3^46^, which have been shown to play a role in chromatin loops but not used in our training. This suggests that loop patterns learned from CTCF ChIA-PET and H3K27ac HiChIP models can be used to predict loops mediated by other factors.

### Peakachu reveals a unique set of short-range interactions

**Fig. 3:**
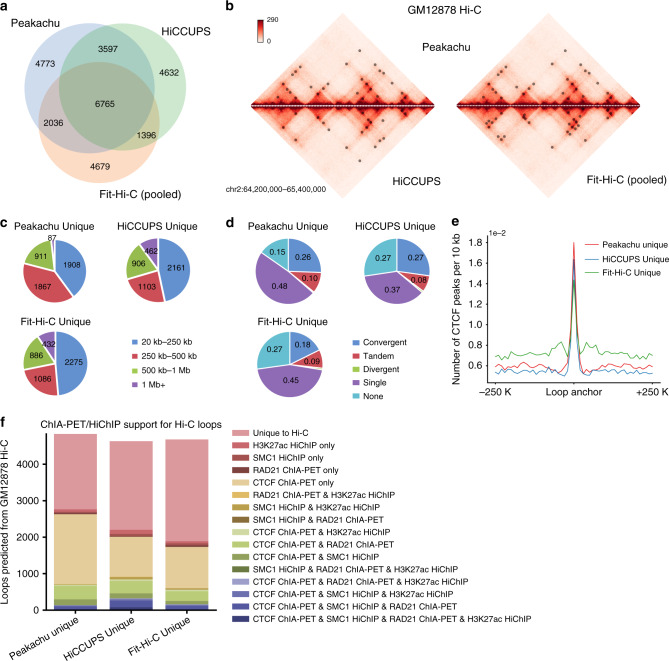
Comparison of Peakachu, HiCCUPS, and Fit-Hi-C in GM12878 Hi-C. **a** Venn diagram of loops predicted by different methods. The original significant interactions from Fit-Hi-C were pooled by the same algorithm used in Peakachu. Parameters of HiCCUPS and Fit-Hi-C were tuned to make the number of loops uniquely predicted by each method similar. **b** Visualization of loops. **c** Distance distribution of loops uniquely detected by each method. **d** CTCF motif patterns of uniquely detected loops by each method. **e** CTCF binding profile surrounding loop anchors. **f** Overlap loops uniquely detected by each method with ChIA-PET/HiChIP interactions. Source data are available in the Source Data file.

To benchmark the performance of Peakachu, we compared it with two current popular enrichment-based methods, HiCCUPS^23^ and Fit-Hi-C^21^ (Fig. 3). First, we ran both methods on the same GM12878 Hi-C matrix at 10 kb resolution. We noticed Fit-Hi-C detected over 120 million significant interactions even with the FDR cutoff <1e-5 (Supplementary Fig. 8a). Therefore, to make a fair comparison, we sorted the Fit-Hi-C outputs by p-values and merged the top 140,000 interactions into 14,876 loops (Fig. 3a, b), with the same pooling algorithm used by Peakachu (Methods). We observed that 72% (12,398 of 17,171) of Peakachu results overlap with either HiCCUPS or Fit-Hi-C predictions (Fig. 3a).

We systematically studied the characteristics of loops uniquely detected by each method. Peakachu-specific predictions contain a higher percentage of short-range loops (~79%, 3775/4773), compared with 70 and 72% for HiCCUPS- and Fit-Hi-C-specific loops (Fig. 3c). As for CTCF binding patterns, we found 85% of Peakachu-specific loops contain active CTCF binding sites at least one anchor, compared with 73% for HiCCUPS and Fit-Hi-C-specific loops (Fig. 3d, e). Most importantly, 58% (2766/4773) of Peakachu-specific loops could be validated by at least one ChIA-PET or HiChIP dataset, while the validation ratios for HiCCUPS specific and Fit-Hi-C-specific loops are 48% (2205/4632) and 40% (1888/4679), respectively (Fig. 3f).

Further, we validated Peakachu-specific loops by comparing them with more external data such as ATAC-Seq, PhyloP conservation scores^47^, and five orthogonal experimental data generated from the 4D Nucleome consortium^48^, including Dilution Hi-C^49^, H3K4me3 PLAC-Seq, RNAPII ChIA-PET, TrAC-loop, and DNA SPRITE^18^. We found anchors of Peakachu-specific loops are enriched with chromatin accessible loci and highly conserved during the evolution, at all genomic distances (Supplementary Fig. 9). APA analysis shows that these Peakachu-specific loops have strong signal enrichment in all five orthogonal datasets (Supplementary Fig. 10), suggesting that Peakachu indeed identified a unique set of chromatin loops at a high validation rate.

We repeated the same aforementioned comparisons in human leukemia cells (K562) and mouse embryonic stem cells (mESC), and found that Peakachu consistently predicted more short-range and CTCF-enriched loops, as well as higher validation rates among orthogonal datasets (Supplementary Figs. 11, 12). Therefore, we conclude that while searching only for the strongest dot signals on a Hi-C map can reveal a large set of chromatin interactions, a supervised machine-learning approach, especially when trained with shorter regulatory interactions, can recover a unique set of chromatin interactions.

### Estimating false discovery rate (FDR) for Peakachu

In order to estimate the FDR of our model, we applied Peakachu to predict loops from a system previously used to investigate the impact of cohesin loss on loop formations^50^. This system used a modified human colorectal carcinoma cell line HCT-116, with an AID domain tagging to both RAD21 alleles, an indispensable component of the cohesin complex. When treated with auxin, RAD21 in this cell line is effectively destroyed, and loops concomitantly disappeared in Hi-C maps genome-wide due to loss of cohesin.

Using a model trained with CTCF ChIA-PET interactions, Peakachu identified only 19 loops genome-wide from the Hi-C map of auxin-treated cells. The same model identified 11,814 loops from the Hi-C map of untreated cells (Supplementary Data 2). Given that the sequencing depths are similar between both maps, we roughly estimated Peakachu’s FDR at ~0.2% (19/11,814).

### Peakachu is robust to sequencing depths

**Fig. 4:**
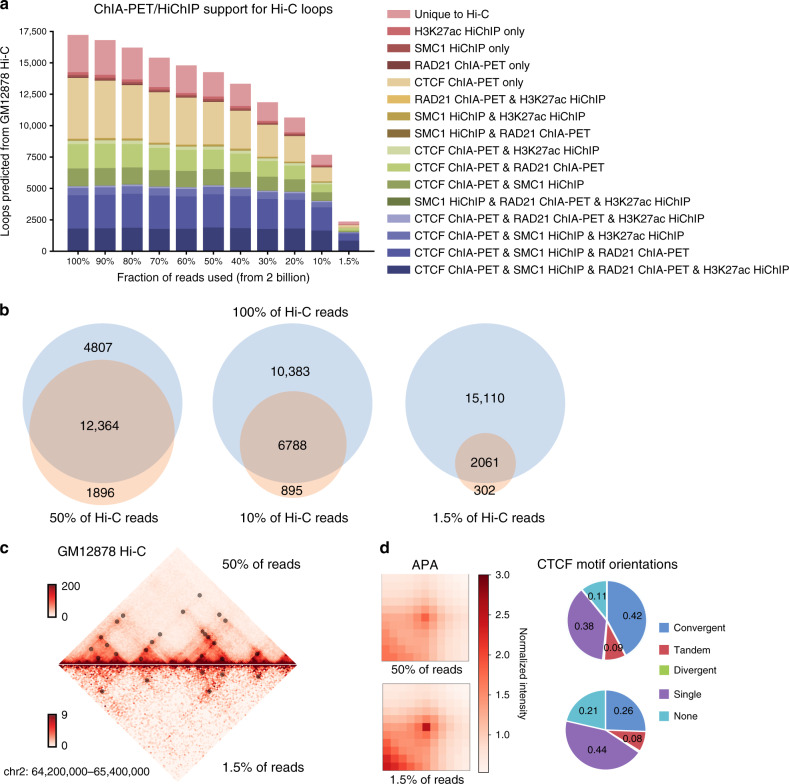
Detection of loops at lower sequencing depths. **a** Predicted interactions from down-sampled versions of the GM12878 Hi-C map with levels of validation by orthogonal methods. **b** Concordance of predictions from datasets with different down-sampled rates. **c** Visualization of a chromosome 2 locus for Hi-C data and predicted loops using 50% and 1.5% of sequencing reads. **d** APA profiles and CTCF motif orientations of 50% and 1.5% loop predictions. The color bar indicates contact counts normalized by the mean of pixels within their windows. Source data are available in the Source Data file.

To test the effect of sequencing depth on the performance of Peakachu, we computationally down-sampled the GM12878 dataset to 11 different depths (ranging from 30 million to 2 billion cis-reads) (Methods), with the same 10 kb resolution for each sequencing depth. We then independently trained and predicted loops in each down-sampled Hi-C matrix. First, we observed that lower sequencing depths generally resulted in reduced numbers of predicted loops (Fig. 4a). However, even at 1.5% down-sample rate (~30 million reads), Peakachu still predict 2363 chromatin loops, 2128 (90%) of which can be validated by at least one orthogonal data. More importantly, we observed that the predicted loops are highly concordant across different sequencing depths, although the models are trained separately. For example, 87% (12,364/14,260) of loops predicted in the 50% down-sampled matrix are also predicted in the original datasets, while 88% (6788/7683) of the loops predicted in the 10% down-sampled matrix are predicted in the original dataset. Even when we used the 1.5% down-sampled matrix, 85% of the predicted loops overlap with loops predicted in the original matrix as well (Fig. 4b, c).

APA analysis of predicted loops at varying sequencing depths showed similar enrichment of contact signals compared to surrounding pixels (Fig. 4d and Supplementary Fig. 13). As sequencing depth decreased, validation rates of predicted loops remained similar while their distance distributions tended toward shorter range (Supplementary Fig. 8b) and retained a majority of loops predicted by HiCCUPS in the same maps (Supplementary Figs. 14, 15).

We noticed that both HiCCUPS and Fit-Hi-C showed more sensitivity to sequencing depths compared with Peakachu (Supplementary Figs. 8a and 14a). For example, at 1.5% down-sampling, HiCCUPS could only identify 655 loops (3.6-fold less than Peakachu) even with the most lenient parameters, and Fit-Hi-C only detected 1,732 interactions after specifying an FDR < 0.1. However, only 79% (519/655) and 83% (1433/1732) of HiCCUPS and Fit-Hi-C loops could be validated by at least one ChIA-PET or HiChIP dataset at this sequencing depth (Supplementary Fig. 16a).

Since we have pretrained 11 models ranging from 30 million to 2 billion read depths, we wanted to investigate whether it is necessary for our users to train a new Peakachu model each time with exactly the same sequencing depths. First, we compared three pretrained models: 100% vs. 90% (2 billion vs 1.8 billion), and 80% vs 90% (1.6 billion vs. 1.8 billion reads). The overlap between each set of prediction is ~85% (Supplementary Fig. 17a); this variation is comparable to the predictions from two biological replicates with similar sequencing depth (14.6% variation, sequencing depth 382 million vs 389 million, Supplementary Fig. 17b). We then evaluated the variation of models trained at more disparate sequencing depths (30 million, 200 million and 1.8 billion) (Supplementary Fig. 17c). We still observed reasonable overlap between models trained from different sequencing depths. Therefore, we believe for any new Hi-C experiments, choosing the pretrained models with the closest sequencing depth will yield satisfactory results.

### Peakachu models are transferrable in different cell types

**Fig. 5:**
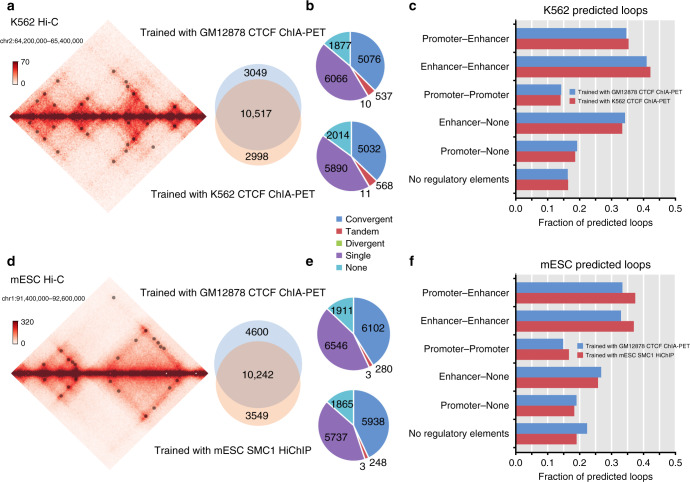
Application of Peakachu in other Hi-C datasets of different tissues and species. **a** Overlap of loops predicted in K562 Hi-C by Peakachu models trained in either GM12878 or K562 data. Both models were trained with CTCF ChIA-PET examples. **b** CTCF binding orientations for predicted loops. **c** Regulatory elements at anchor loci of predicted loops. **d**–**f** Repeat analysis for loops predicted in mESC Hi-C by Peakachu models trained with either GM12878 CTCF ChIA-PET or mESC SMC1 HiChIP. Source data are available in the Source Data file.

To test whether models trained in one cell type can be applied in other cell types, we first used the model trained in GM12878 CTCF ChIA-PET to predict loops in the human chronic lymphocytic leukemia cell line K562 (~500 million *cis*-reads)^8^ and in mouse embryonic stem cells (1.9 billion *cis-*reads)^51^. To match the sequencing depths in K562 and mESC, we used models trained with 20% and 90% GM12878 Hi-C reads and CTCF ChIA-PET examples (Fig. 5). In K562, we predicted 13,566 chromatin loops: 37% (5,076/13,566) of which contain convergent CTCF binding sites and 41% (5623/13,566) with CTCF binding at both anchors, and an additional 45% (6066 loops) having CTCF binding at one anchor (Fig. 5b). We predicted 14,842 loops in mESC: 41% (6102 of 14,842) contained convergent CTCF binding sites and an additional 44% (6546 of 14,842) had one CTCF anchor (Fig. 5e). Both sets of predictions contained regulatory elements in at least 80% of candidate loops (Fig. 5c, f).

Next, we compared predicted loops in K562 from the model trained with GM12878 CTCF ChIA-PET with a model trained with K562 CTCF ChIA-PET^52^. Overall, the predictions are highly similar. A total of 13,566 candidate loops were predicted by the GM12878 model vs 13,515 by K562 model. 78% (10,571/13,566) of GM12878 model and 78% (10,571/13,515) of loops from the K562 model are the same. Their percentage of convergent CTCF binding sites and percentage of enrichment of *cis*-regulatory elements are consequently similar as well (Fig. 5b, c).

To further validate the transferability of Peakachu models, we compared loops predicted in mESC by models trained with either GM12878 CTCF ChIA-PET or mESC SMC1 HiChIP^13^. Again, the total number of predictions was similar, with 14,842 candidate loops from the GM12878 model and 13,791 from the mESC model. Of these, 10,242 were the same, representing 69% of predictions from the GM12878 model and 74% of those from the mESC model (Fig. 5d). While the total overlap was slightly less than the comparable K562 analysis, we found that both sets of mESC predictions had similar distributions for both CTCF binding site orientations (Fig. 5e), and that regulatory elements were slightly more enriched in the model trained with mESC SMC1 HiChIP (Fig. 5f).

With K562 and mESC loops serving as a proof-of-concept for transferable GM12878-trained models in other cell types and species, we next predicted interactions in 53 additional Hi-C datasets ranging from 25 million to 3.6 billion *cis-*reads using models trained with down-sampled GM12878 contact maps (Supplementary Data 2). We found that the majority of the predicted loops (>87.5%) are located within the same TADs (Supplementary Fig. 18) and APA analysis shows there are strong enrichment of Hi-C signals for the loops predicted in all the datasets (Supplementary Fig. 19). Furthermore, to investigate whether differential chromatin loops are associated with differential gene expression, we first compared loops predicted in GM12878 and K562 cells. By requiring at least 2-fold changes in the Peakachu probability score for a loop, we identified 1134 GM12878-specific loops and 1075 K562-specific loops (Supplementary Fig. 20). Interestingly, we found that genes located in GM12878-specific loops were also expressed at a higher level in GM12878, while genes in K562-specific loops had higher expression level in K562 cells. We also performed a similar analysis between GM12878 and IMR90, and made a similar observation (Supplementary Fig. 20). These results suggest loops predicted by Peakachu are closely related to gene regulations and biological functions.

### Applying Peakachu on DNA SPRITE and Micro-C data

**Fig. 6:**
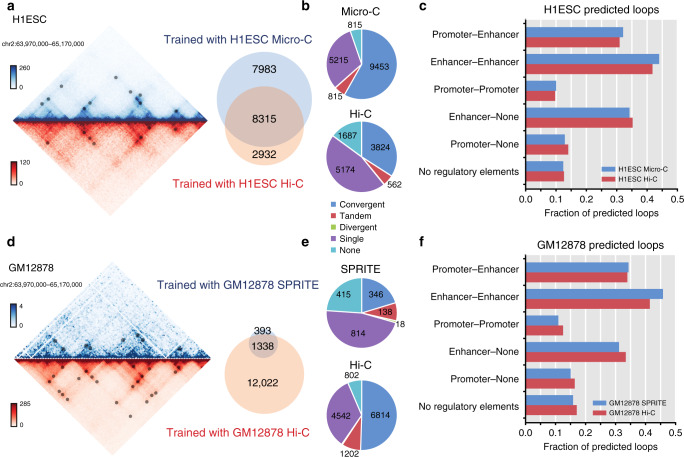
Cross-platform comparison of Peakachu loops in different tissue types. **a** Overlap of loops predicted in H1ESC by Micro-C and Hi-C, using the same CTCF ChIA-PET training set. **b** Patterns of CTCF binding site orientations in predicted loops. **c** Regulatory elements at anchor loci of predicted loops. **d**–**f** Repeat analysis comparing DNA SPRITE with Hi-C in GM12878, using the same CTCF ChIA-PET training set. Source data are available in the Source Data file.

We were interested in Peakachu’s potential to perform cross-platform comparisons. To this end, we tested the performance of Peakachu in Micro-C^10^, a variant of Hi-C protocol capable of higher contact resolutions, and DNA SPRITE^18^, which interrogates chromatin interactions by using a split-pool procedure and assigning a unique sequence barcode for each chromatin contact. We downloaded the H1-ESC Hi-C and Micro-C data^53^ from the 4DN data portal and the GM12878 DNA SPRITE data from Quinodoz et al.^18^. In both cell lines, CTCF ChIA-PET data are available and were used as positive training sets for Peakachu models trained for Hi-C, Micro-C, and SPRITE (Fig. 6). We used the same parameters and search space for all analyses.

In Micro-C, we predicted a total of 16,298 loops, which is higher than the number of loops we predicted in Hi-C data (11,247). This is potentially due to the fact that Micro-C improved upon the Hi-C protocol by using a different crosslinker and fragmenting chromatin with higher-resolution enzyme MNase^10^. 74% (8315/11,247) of the Hi-C loops and 51% of the Micro-C loops are the same (Fig. 6a). Interestingly, the Micro-C predicted loops contain a higher convergent CTCF ratio (58% vs 34%, Fig. 6b) and in general, a higher CTCF binding rate. Micro-C predicted loops also contain a slightly higher percentage of enhancer-promoter or enhancer-enhancer interactions (Fig. 6c).

The DNA SPRITE dataset for GM12878 contains 135 million *cis-*reads, resulting in a contact map that was quite sparse compared to the Hi-C map comprised by 2 billion reads. To the best of our knowledge, we are not aware of any successful effort that can identify loops from this set of SPRITE data. Here by training and applying the Peakachu model, we were able to predict 1731 loops, 77% (1338/1731) of which can be validated by Hi-C predicted loops (Fig. 6d). The majority of SPRITE loops in this dataset (66%, 1140 of 1731) are within 250 kb, potentially due to the sparsity of the contact map. Compared with Hi-C, loops identified in SPRITE have lower convergent CTCF ratios (Fig. 6e) but have similar percentages of interactions between cis-regulatory elements (Fig. 6f). To further validate the SPRITE loops predicted by Peakachu, we compared them with four orthogonal types of data, including CTCF ChIA-PET, RAD21 ChIA-PET, SMC1 HiChIP, and H3K27ac HiChIP datasets, and found that 85% (1475/1731) of SPRITE loops could be supported by at least one source (Supplementary Fig. 21).

Overall, these analyses show that a general data-driven framework can produce viable decision functions to classify loops in a platform-agnostic manner, especially for deeply sequenced contact maps.

## Discussion

Here we present Peakachu, a machine-learning framework to predict chromatin loops from genome-wide contact maps. To the best of our knowledge, all current loop detection algorithms are based on searching for statistically enriched interactions against a global or local background, and vary in choices of statistical model and background definitions^21–29^. By learning from enrichment-based platforms such as ChIA-PET/HiChIP or Capture Hi-C, Peakachu can detect high-quality loop interactions from genome-wide interaction data such as Hi-C and SPRITE, even at low sequencing depths.

In total, we tested five sets of interactions to train our model, including CTCF ChIA-PET, RAD21 ChIA-PET, Smc1 HiChIP, H3K27ac HiChIP, and promoter Capture Hi-C (Supplementary Figs. 22 and 23). We noted that the CTCF ChIA-PET model predicts more long-range loops, while the H3K27ac HiChIP model predicts more short-range loops which link distal regulatory elements to their potential target genes. Examining the Random Forest feature importance, we found that the most important predictor in a CTCF model is the center pixel, while the H3K27ac model is additionally driven by lower-left pixels (Supplementary Fig. 24). We also noted that although Capture Hi-C is different from antibody-based assays, the models trained with Capture Hi-C also performs well and the results are comparable to models trained with ChIA-PET and HiChIP data (Supplementary Figs. 22 and 23), suggesting that Peakachu is robust to different types of positive training dataset.

Further, one potential extension of this framework will be training with interactions from more orthogonal data types for the study of higher-order chromatin organization, such as HiFISH imaging data. To evaluate how many positive training data points needed to train a Peakachu model, we manually picked ~200 annotated loops (Supplementary Data 3) and found it was enough for Peakachu to train a model to perform genome-wide predictions (Supplementary Fig. 25), indicating that Peakachu is readily applicable to train a working model with only several hundred positive data points.

Since there have been data augmentation methods^38,54^ to enhance the data resolution of Hi-C data, we evaluated whether they can help further improve the performance of Peakachu. For this purpose, we first down-sampled GM128787 Hi-C matrix at 1.5 and 10%, and then enhanced them genome-wide with Boost-HiC^54^, a method based on detecting the shortest path on the contact graph. We observed that Boost-HiC can help Peakachu identify more loops compared with un-boosted Hi-C matrices (Supplementary Fig. 16). Depending on the augmentation rate, the overall validation rate of loops from boosted Hi-C matrices can be lower, which may be a reasonable trade-off depending on the design of the study.

By comparing the performance of Peakachu, HiCCUPS, and Fit-Hi-C, we found that although a large proportion of predictions are shared by all three methods, each method detects a unique set of chromatin loops with various distance distributions, CTCF binding, and validation ratios by orthogonal datasets. This result suggests that the complete set of chromatin loops might not follow the same interaction pattern and be captured by a single method. The major improvement of Peakachu is its robustness to sequencing depth, which makes it applicable for predicting chromatin loops in Hi-C data with only ~30 million intrachromosomal reads.

The number of techniques for chromosome conformation study continues to grow, and there are still platforms such as DNA SPRITE that lack dedicated algorithms for chromatin loop detection. Here we show the generalizability of our framework by demonstrating its performance in a DNA SPRITE data matrix. In future studies, we will apply Peakachu framework in more available platforms to investigate advantages and pitfalls of each technique in loop detecting and hope to unveil the complete picture of loop-level structure in mammalian genomes.

## Methods

### Peakachu Framework

Fitting a Peakachu model requires two components: a Hi-C matrix binned to 10 kb and an interaction list that defines a positive training set. For every interaction in the list, a corresponding 11 × 11 window centered at the interaction is collected from the Hi-C matrix. The ratio of the center pixel to the lower-left quadrant (P2LL) of this window is used as an indicator variable prior to training, and the minimum P2LL for the positive class is set to 0.1. In other words, samples from the input training list are rejected if their Hi-C value is less than 10% of the average value within the loop. After collecting the positive class, a comparable number of windows with random coordinates and nonzero centers are collected to define a negative class.

Each sample is decomposed into a vector of 2*n* + 1 features, where *n* is the radius of a sample’s feature space. With *n* = 5, 243 features are constructed from 11×11 windows. One hundred and twenty-one of these represent the values of each pixel in an 11 × 11 window. Another 121 represent the relative ranks of each pixel within the window. A final variable, P2LL, is appended to each vector of features. Using scikit-learn, the training set is then used as input to fit a random forest of 100 decision trees and each tree is trained on a random combination of 15–20 features. To avoid the overfitting problem, the whole dataset is split into a separate training and test dataset in a chromosome-wise manner, i.e., 22 chromosomes are used to train a model that is used to make final predictions in 1 hold-out chromosome. Therefore, the final predicted loops would never be used/seen during training. During the training, we use a 3-fold cross-validation grid search to find the optimal hyperparameters (including splitting criterion, maximum tree depth and class weights) that yield a satisfying generalization performance. Matthew’s Correlation Coefficient is used as the primary metric for selecting the best model.

Trained models can be applied to contact maps of the same platform as the training matrix. Peakachu defines feature vectors for all nonzero pixels within a given genomic span, and scores each using the predict_proba method provided by the scikit-learn library. Usually, highly scored pixels are found grouped together, and only one representative pixel is reported from each cluster. To select representative pixels, we developed a greedy algorithm entailing two steps: first, define 1D loop anchor regions enriched for highly scored (*P* > 0.9) pixels, then run DBSCAN between any two connected anchor regions. The identification of the loop-enriched anchors was performed by counting the candidate pixels and finding peaks along the chromosomes. Specifically, we applied the find_peaks and peak_widths functions from Python’s Scipy package to locate the peak summits and estimate the peak widths, respectively.

### Performance test of different machine-learning frameworks

To train a model best separating defined training sets, we tested 6 machine-learning frameworks implemented in scikit-learn: Gaussian Naïve Bayes, Perceptron, Logistic Regression, SVM (linear kernel), SVM (rbf kernel) and Random Forest. All frameworks used a similar strategy described above to train a model for each chromosome independently using data from the rest of the 22 chromosomes. Except for Gaussian Naïve Bayes, we used the same grid search approach to find the optimal hyperparameters for all frameworks during the training. Below are hyperparameters tuned for each framework:

1. Perceptron: penalty/regularization and class weights
2. Logistic Regression: penalty, C (inverse of regularization strength), class weights and the Elastic-Net mixing parameter
3. SVM (linear kernel): the loss function, C and class weights
4. SVM (rbf kernel): C and class weights
5. Random Forest: splitting criterion, maximum tree depth and class weights

The prediction performance of each framework was measured by Matthews Correlation Coefficient (MCC), Accuracy (ACC), Receiver Operating Characteristic (ROC) Curves and Area Under the ROC curve (AUC). As shown in the Supplementary Fig. 27, Random Forest achieved best prediction performance and consumed relatively less training time for both CTCF and H3K27ac training sets used in this study.

### Peakachu probability tuning

Since Peakachu models are standard random forests, the assigned probability value for each pixel can be used as a filtering criterion. Lowering the probability threshold always generates more loops with less ChIA-PET/HiChIP dataset support, while higher probability thresholds achieve fewer but better-quality loops. For most predictions in GM12878 of this work, we set the cutoff to 0.97 and 0.92 for the CTCF model and the H3K27ac model, respectively. In Supplementary Fig. 17a, b, the probability was tuned to obtain a similar number of loops as the model trained with 90% of Hi-C reads. Similarly, in Supplementary Fig. 17c, the probability for the models trained with 90% or 1.5% of Hi-C reads was tuned according to the model trained with 10% of Hi-C reads. The cutoffs used in other cell lines are detailed in Supplementary Data 2.

### Loop detection with HiCCUPS and Fit-Hi-C

We used four kind of HiCCUPS settings in this work: (1) When comparing with Peakachu and Fit-Hi-C, we tuned the −f and −t parameters to make the number of loops uniquely predicted by each method similar. (2) When testing HiCCUPS performance on the down-sampled contact maps, we first ran HiCCUPS with the default parameters (Supplementary Fig. 14), which generally outputted less than 1/3 of the Peakachu loops at each down-sample rate. (3) We also tuned the parameters to make HiCCUPS detect a similar number of Peakachu loops with 100% Hi-C reads and then applied identical parameters (−f 0.37 −t 1.4,1,1,1) to the rest of the down-sample rates (Supplementary Fig. 15). (4) At the 1.5% down-sample rate (~30 million reads), no loops could be detected even with the lenient parameters used in (3); therefore, we further tuned the parameters to −f 0.9 −t 3.6,1,1,1 and identified 655 loops (Supplementary Fig. 16).

Fit-Hi-C was run on the 10 kb Hi-C matrices with the following settings: −p 2 −m 10 −U 3,000,000 -L 50,000. The results of the 2nd spline pass were then filtered with the q-value cutoff < 1e-5 (Supplementary Fig. 8a). To make a fair comparison with Peakachu and HiCCUPS on the 100% matrix, we sorted the detected interactions by p-values and performed the same pooling algorithm used by Peakachu. At the 1.5% down-sample rate, we changed the q-value cutoff to 0.1 because no interactions remained with q-value < 1e-5.

### Down-sample Hi-C reads to a specified ratio

The contact maps were down-sampled using a binomial probability without re-mapping. For the down-sample rate *α* (0<*α<1*), we iterated each nonzero pixel in the full contact matrix **M**_*ij*_ and designated the count frequency a random integer number generated from a binomial distribution of parameters **M**_*ij*_ and *α*, where **M**_*ij*_ is the contact count of the 100% Hi-C matrix between bin *i* and bin *j*.

### Enrichment analysis of TFs and histone modifications

To validate that Peakachu loops can also involve factors other than CTCF and H3K27ac, we downloaded the ENCODE ChIP-Seq peak files for 133 transcription factors (TFs) and 10 histone modifications in GM12878. Then a fold enrichment score was calculated for each TF or histone modification at loop anchors. Briefly, we first identified non-redundant loop anchors from Peakachu-predicted loops in GM12878. For each TF or histone modification, we iterated this anchor list and counted the number of anchors that overlapped at least one ChIP-Seq peak. Then we randomly shuffled the loops to generate 50 controls and repeated the same procedure for each control. For every control, the genomic distance distribution and the number of random loops on each chromosome stayed the same, and the interval between the two ends of each random loop did not overlap any gaps in the reference genome (hg19). Finally, the fold enrichment score was calculated by dividing the number of anchors containing ChIP-Seq peaks by the average number of random loci containing ChIP-Seq peaks (Fig. 2f).

### Reporting summary

Further information on research design is available in the Nature Research Reporting Summary linked to this article.

## Supplementary information

Supplementary Information

Description of Additional Supplementary Files

Supplementary Data 1

Supplementary Data 2

Supplementary Data 3

Reporting Summary

## Data Availability

All datasets used in this work are summarized in Supplementary Data 1. The Hi-C contact maps of GM12878 and K562 were obtained from ftp://cooler.csail.mit.edu/coolers/hg19/. The DNA SPRITE contact map for GM12878 was obtained from 4DN data portal with accession code 4DNFIUOOYQC3. The Hi-C contact map of H1-ESC was obtained from 4DN data portal with accession code 4DNFI6HDY7WZ. The Micro-C contact map of H1-ESC was obtained from 4DN data portal with accession code 4DNFI9GMP2J8. The CTCF ChIA-PET interactions in GM12878 were obtained from Tang et al.^24^. The Rad21 ChIA-PET interactions in GM12878 were obtained from Heidari et al.^39^. The SMC1 HiChIP interactions in GM12878 were obtained from Mumbach et al.^13^. The H3K27ac HiChIP interactions in GM12878 were obtained from Mumbach et al.^40^. The promoter Capture Hi-C interactions in GM12878 were obtained from Cairns et al.^28^. The CTCF ChIA-PET interactions in K562 were obtained from ENCODE with accession code ENCFF001THV. The SMC1 HiChIP interactions in mouse ESC were obtained from Mumbach et al.^13^. The CTCF ChIA-PET interactions in H1-ESC were obtained from 4DN data portal with accession code 4DNESR9S8R38. All aforementioned positive training datasets can be found at https://github.com/tariks/peakachu/tree/master/training-sets. The enhancer and promoter loci in GM12878, K562, H1-ESC, and mouse ESC were extracted from public ChromHMM annotations in ENCODE and can be found at https://github.com/tariks/peakachu/tree/master/analysis/annotations. The genome-wide CTCF motifs in human and mouse were obtained from https://bcm.app.box.com/v/juicerawsmirror/folder/11363582187. The predicted chromatin loops in 56 Hi-C datasets can be downloaded from the 3D Genome Browser (http://3dgenome.org). Source data are provided with this paper.

## Source data

Source Data

## Code availability

We used cooler 0.8.5 to store and extract contact matrices. We used Juicer 1.11.09 for HiCCUPS loop detections. For Fit-Hi-C, we used the version 2.0.7. We used deeptools 3.1.3 to generate the heatmaps in Supplementary Fig. 7. We used hicpeaks 0.3.4 to generate APA plots. The peakachu source code, the down-sampling script, the interaction pooling scripts and the pretrained models are available in GitHub under the MIT License: https://github.com/tariks/peakachu.
