# Supplementary material for: A supervised learning framework for chromatin loop detection in genome-wide contact maps
Source: Nat Commun. 2020 Jul 9;11:3428. doi: 10.1038/s41467-020-17239-9 (PMC7347923; doi:10.1038/s41467-020-17239-9)
Supplement: Supplementary file 1 — Supplementary Information [file 41467_2020_17239_MOESM1_ESM.pdf]

Supplementary Information

A supervised learning framework for chromatin loop detection in genome-wide contact maps

Salameh and Wang et al.

Supplementary Figures

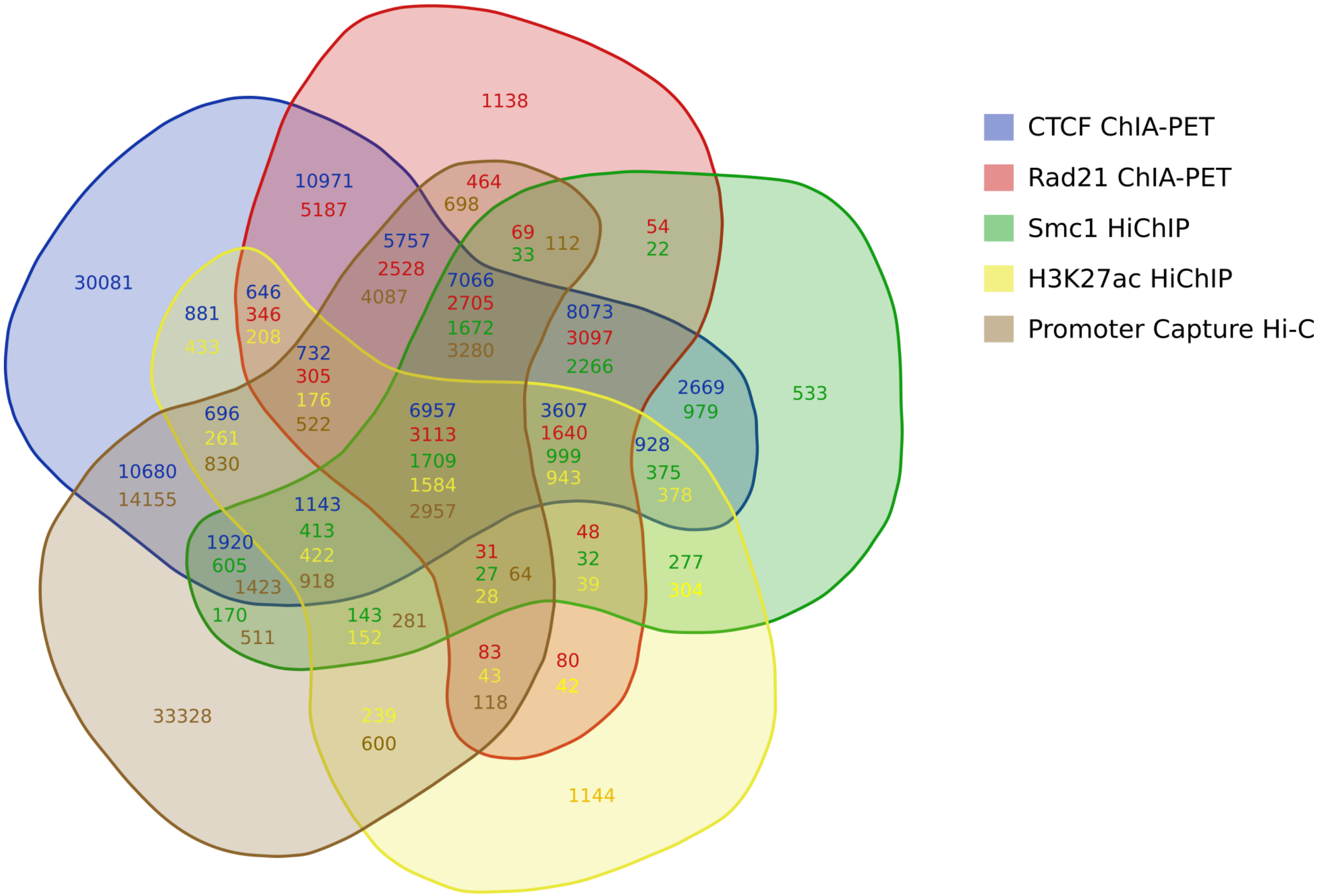

Supplementary Figure 1 | Venn diagram of positive training sets in GM12878. Overlaps were computed with the bedtools pairtopair command with parameters –slop 15000 –type both. In overlap regions, the contribution of each dataset is coded by color and order of the legend.

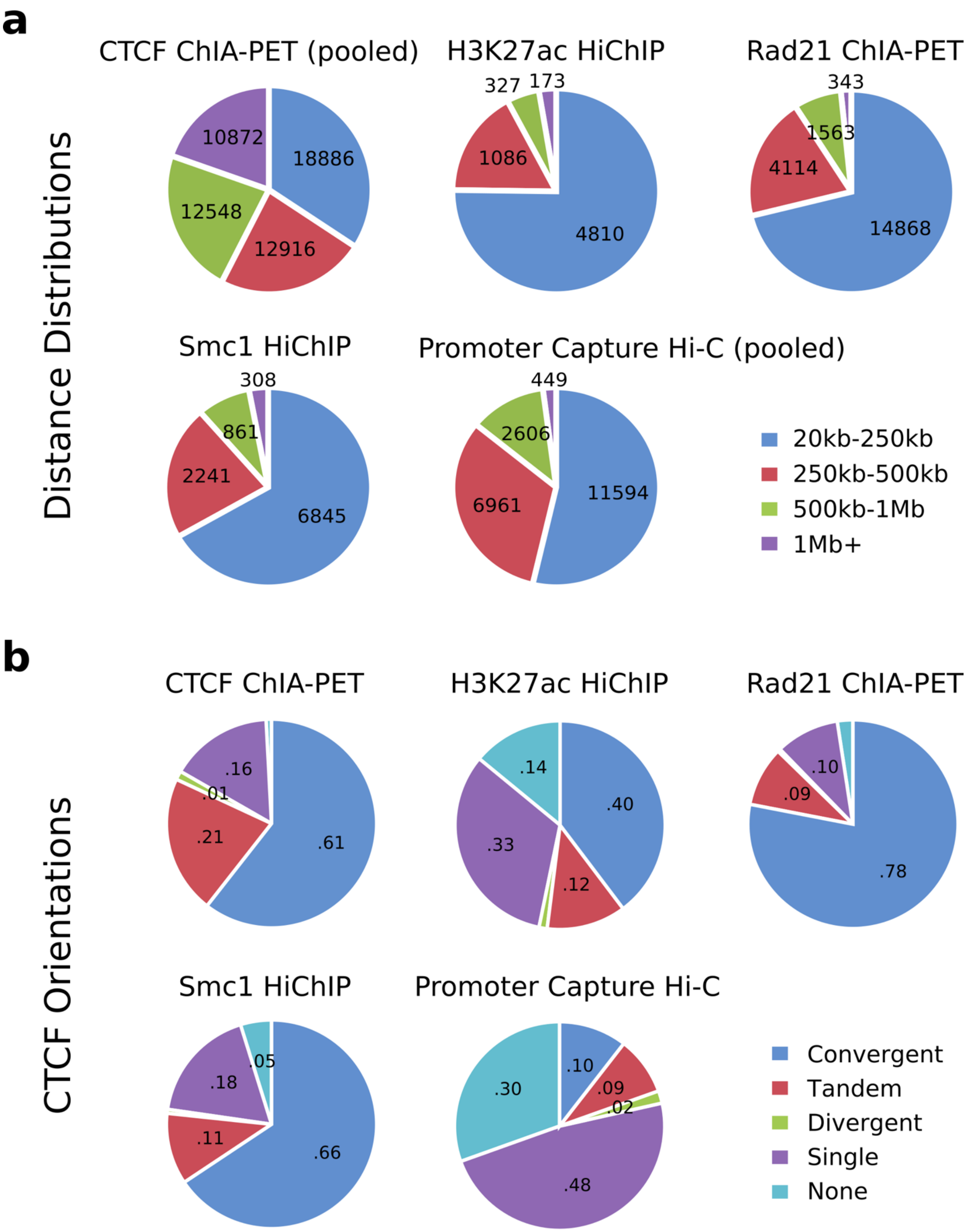

Supplementary Figure 2 | Distance distributions and CTCF binding patterns of 5 positive training sets in GM12878. **a**. Pie charts for distance distributions. Original CTCF ChIA-PET and promoter capture Hi-C interactions were pooled with the same algorithm used in Peakachu to remove local redundancy. **b**. Pie charts for CTCF binding patterns.

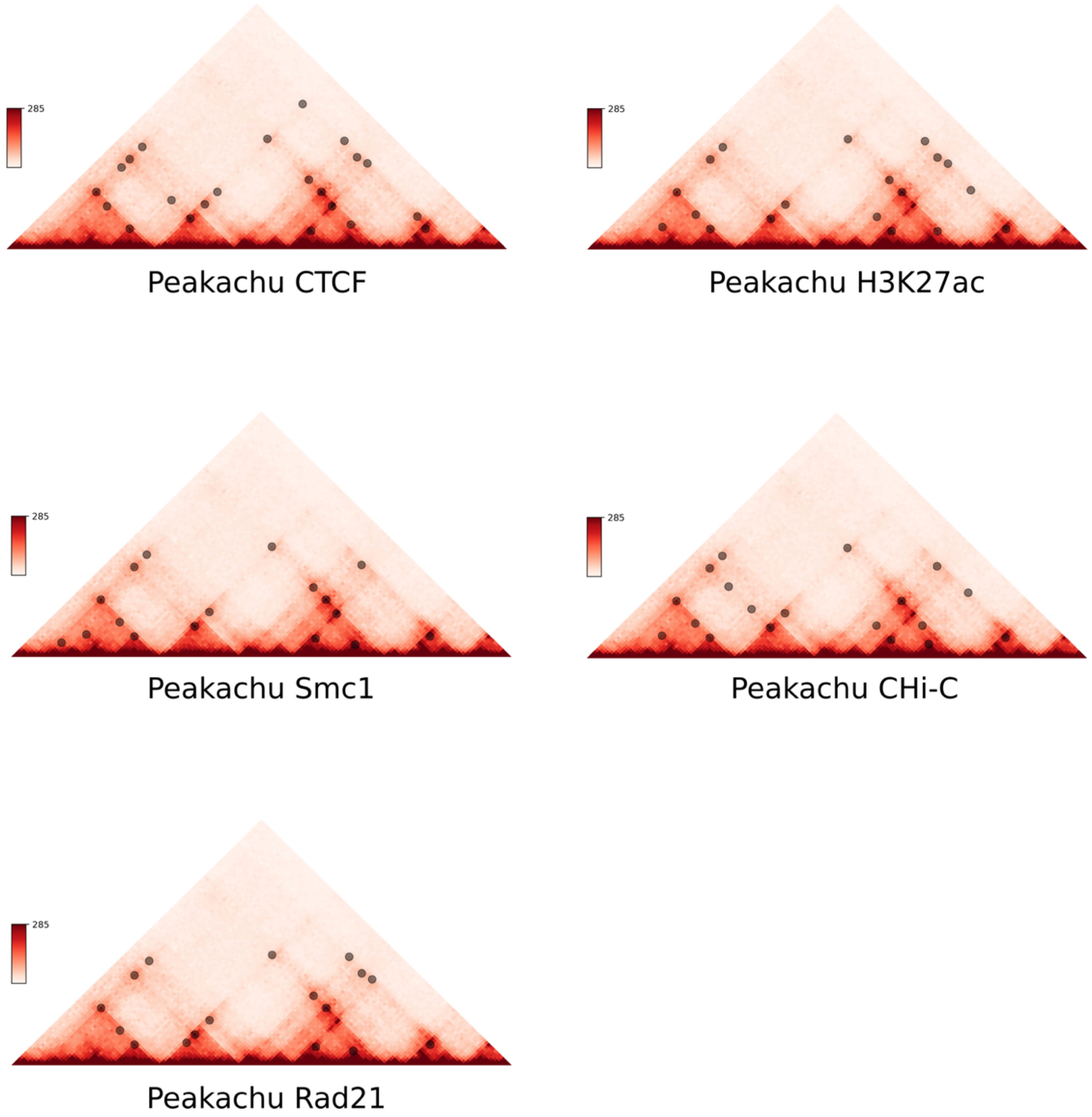

Supplementary Figure 3 | Screenshots showing loop predictions of the same region (chr2: 64,200,000 – 65,400,000) in GM12878 Hi-C by models trained with different orthogonal datasets. Source data are available in the Source Data file.

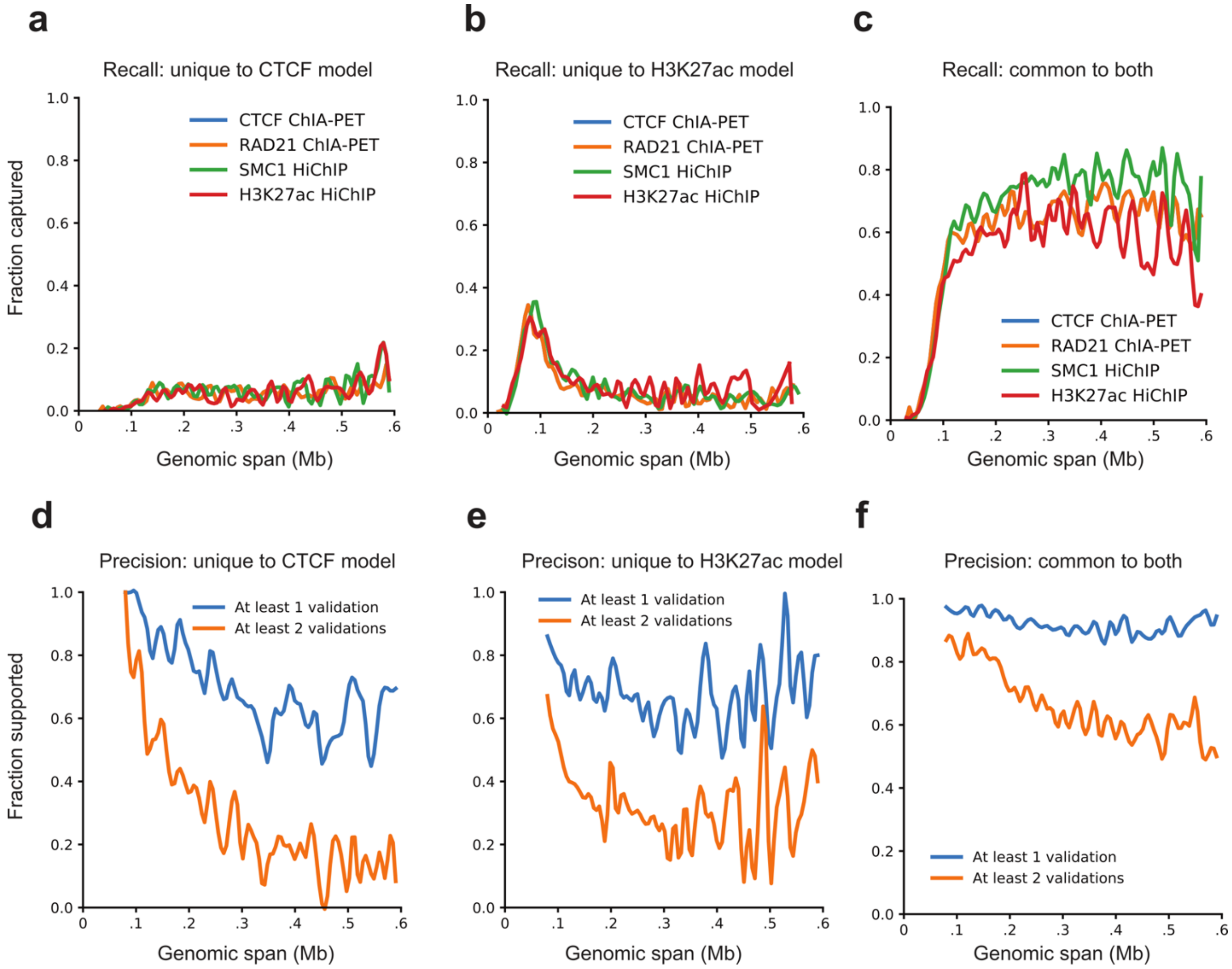

Supplementary Figure 4 | Recall and precision of predictions made by Peakachu models trained with different inputs in GM12878. a-c. Interactions from orthogonal datasets recaptured from Hi-C predictions by two Peakachu models. **d-f.** Ratio of loops with orthogonal support among 4,225 unique predictions from the CTCF model, 4,183 unique predictions from the H3K27ac model, and 9,135 loops predicted by both models. Source data are available in the Source Data file.

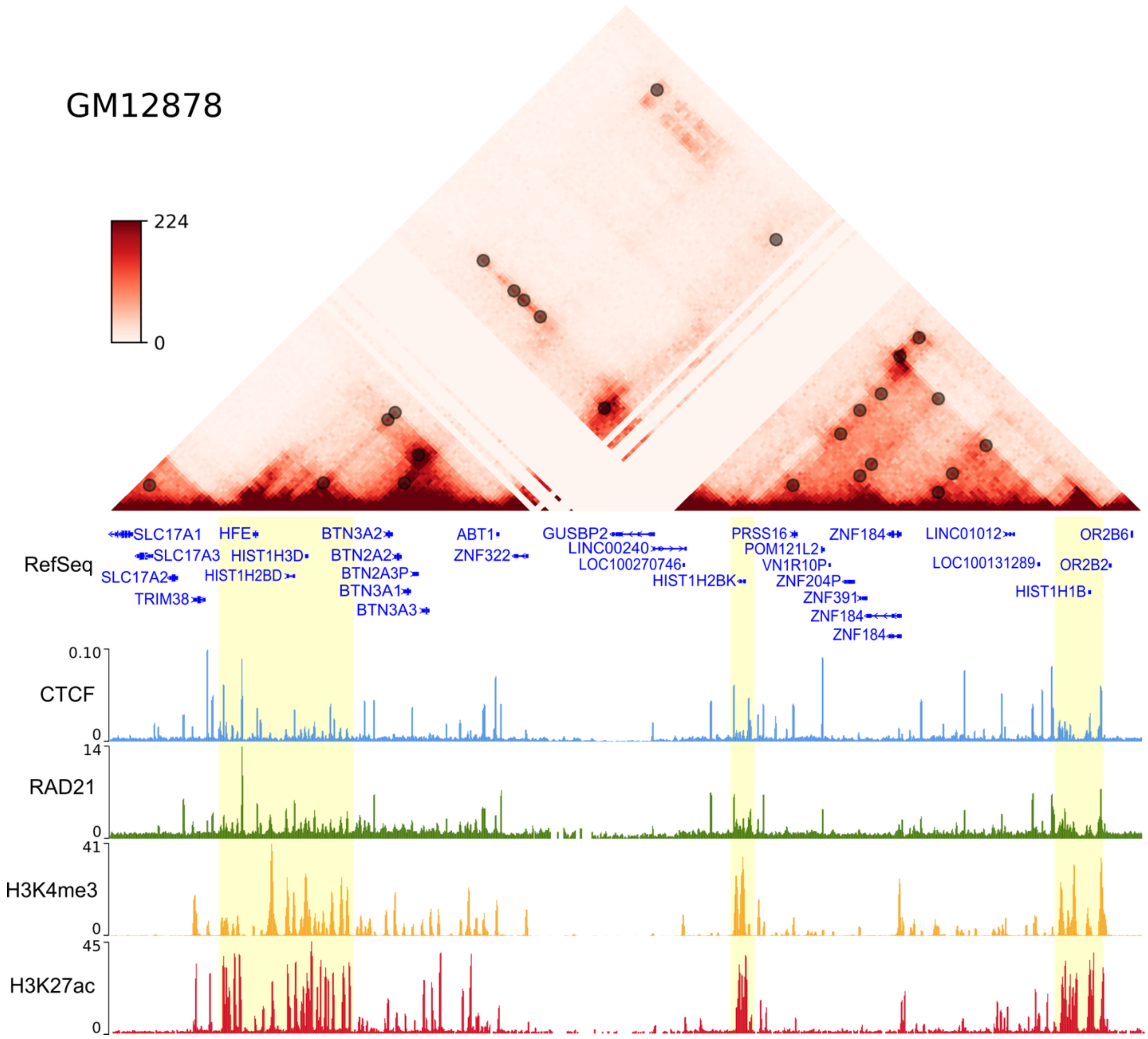

Supplementary Figure 5 | Peakachu predictions capture loops between *HIST1* gene clusters in GM12878. The region (chr6: 25,790,000 – 27,950,000) is shown. Below the Hi-C map are CTCF, RAD21, H3K4me3 and H3K27ac ChIP-Seq signal tracks from ENCODE.

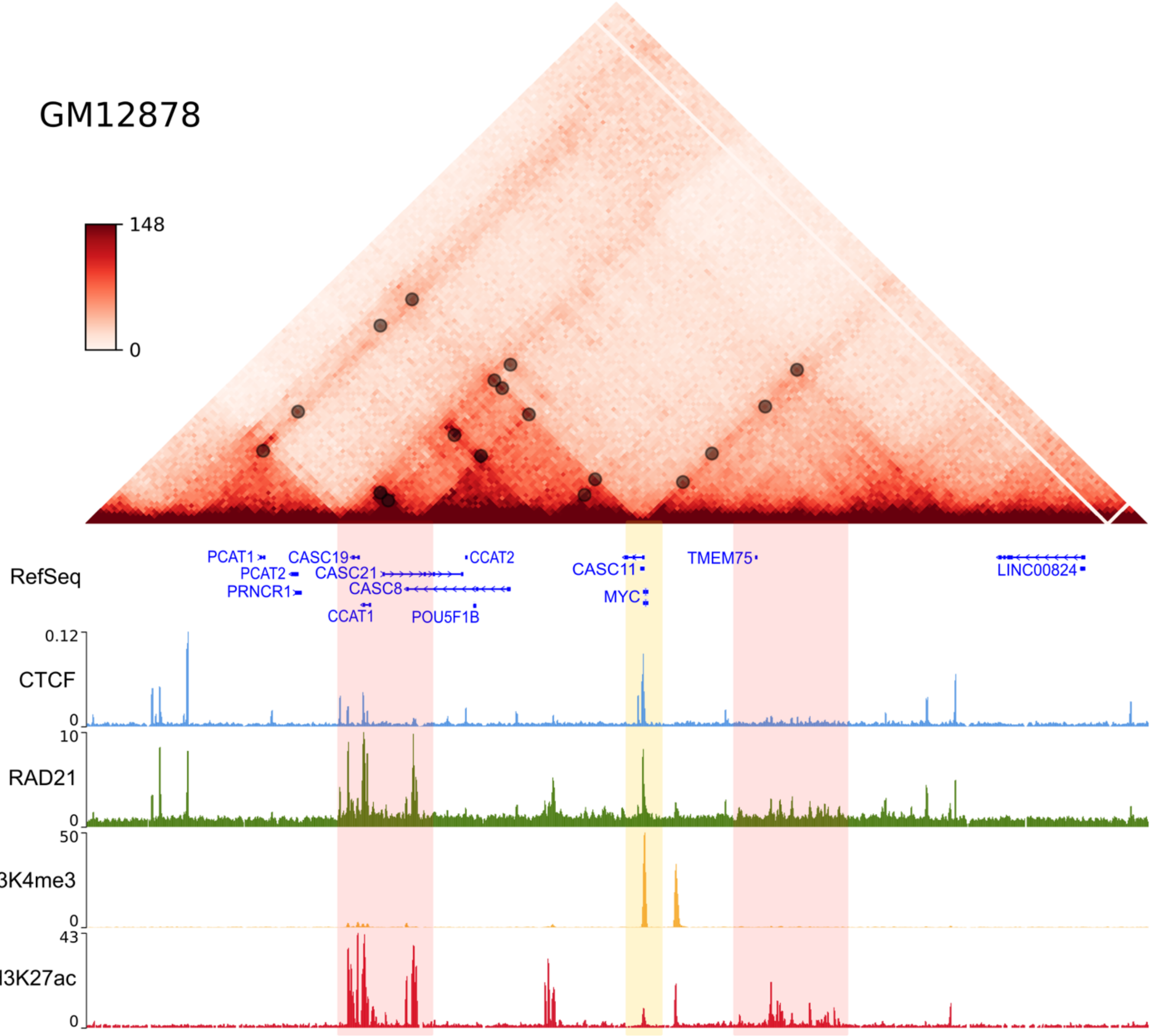

Supplementary Figure 6 | Peakachu predictions capture loops between the *MYC* gene and distal enhancers in GM12878. The Hi-C map and ChIP-Seq signal tracks are aligned for the region (chr8: 127,700,000 – 129,700,000).

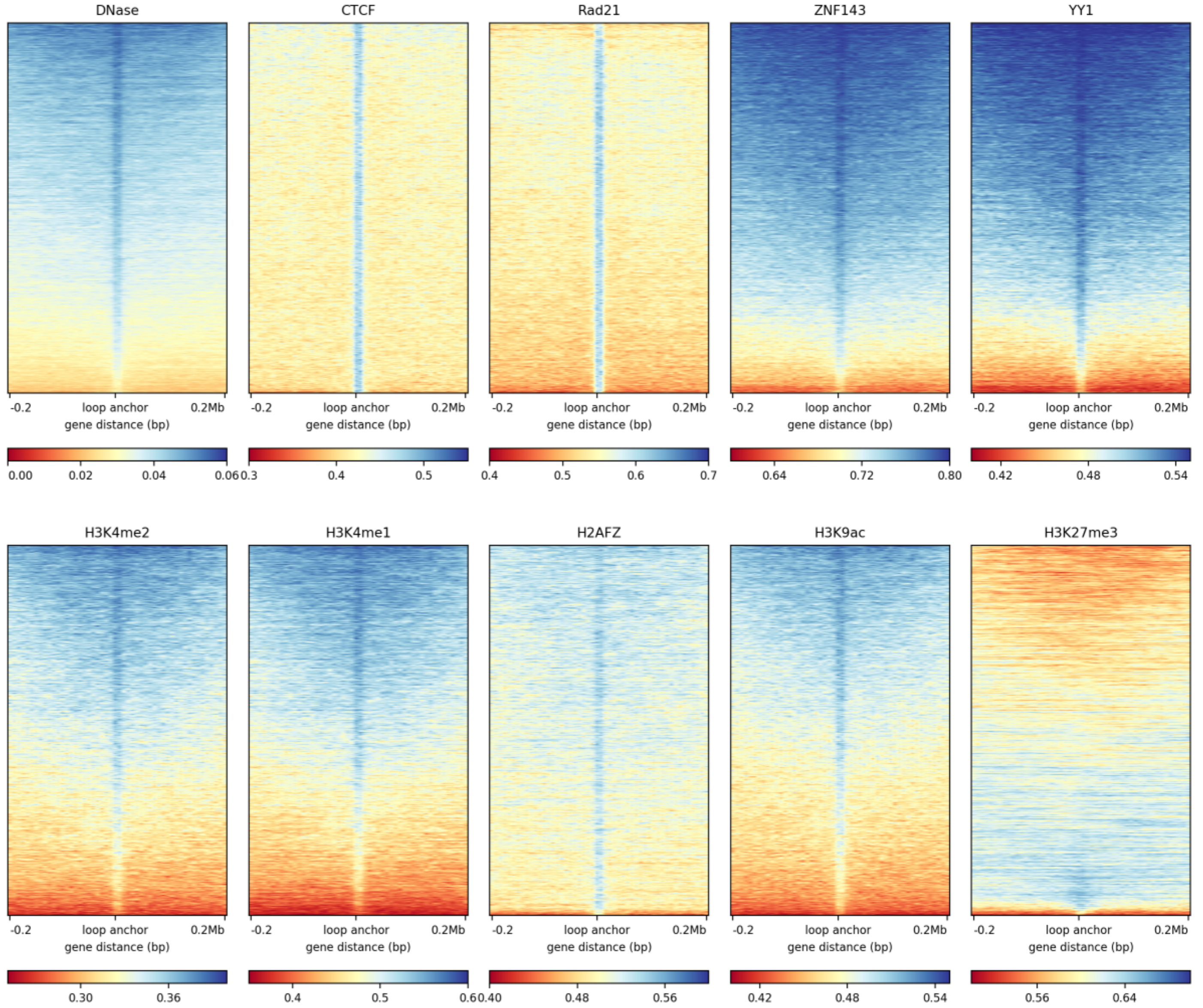

Supplementary Figure 7 | Detailed DNase-Seq and ChIP-Seq signals across 22,817 Peakachu loop anchors in GM12878. The heatmaps were generated by deepTools.

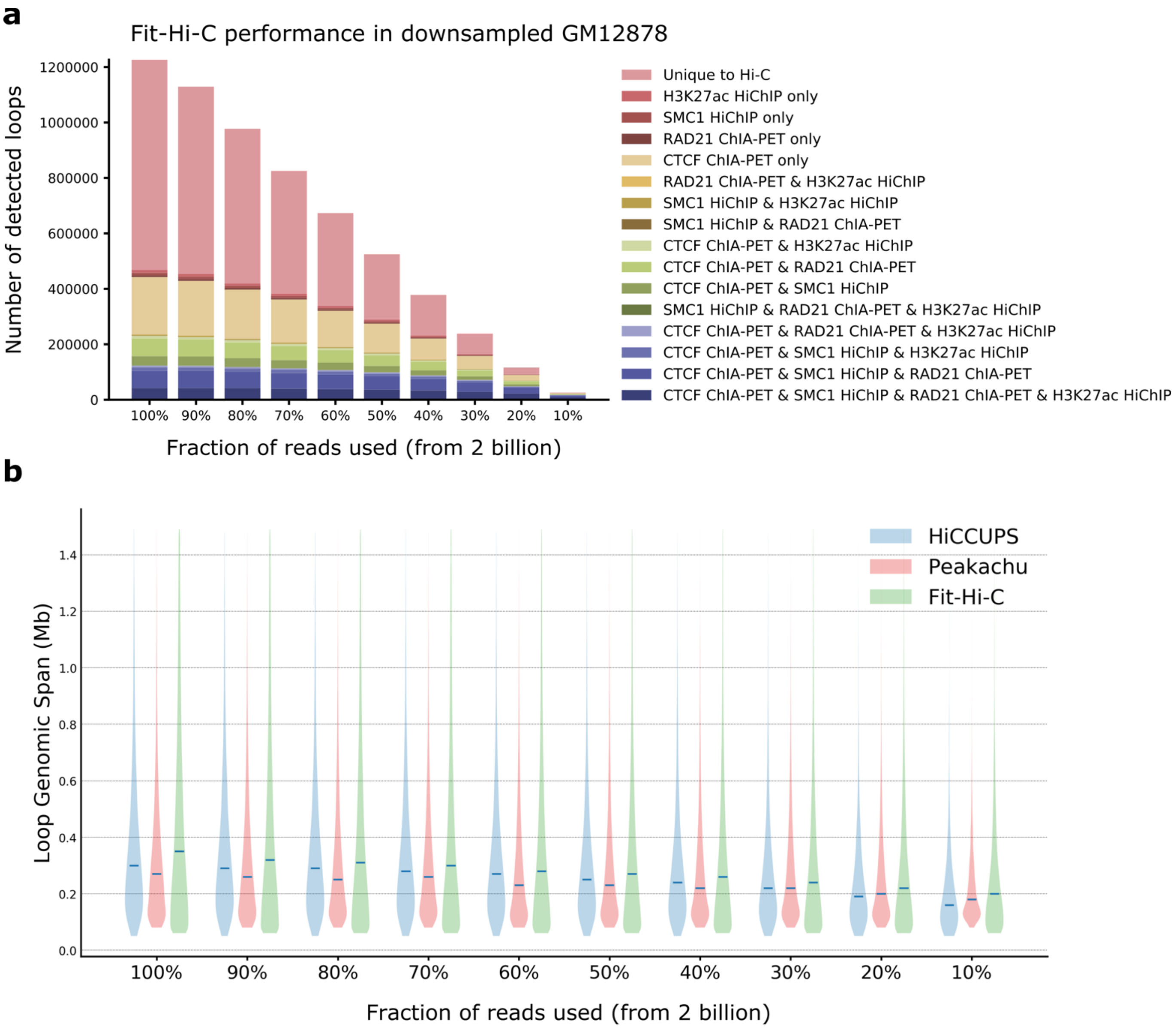

Supplementary Figure 8 | Performance of Fit-Hi-C for down-sampled GM12878 contact maps. a. Overlap of Fit-Hi-C interactions with ChIA-PET and HiChIP datasets in GM12878 at different read depths. The q-value cutoff was set to 1e-5. **b.** Distance distributions of Peakachu, HiCCUPS and Fit-Hi-C loops at different read depths. Source data are available in the Source Data file.

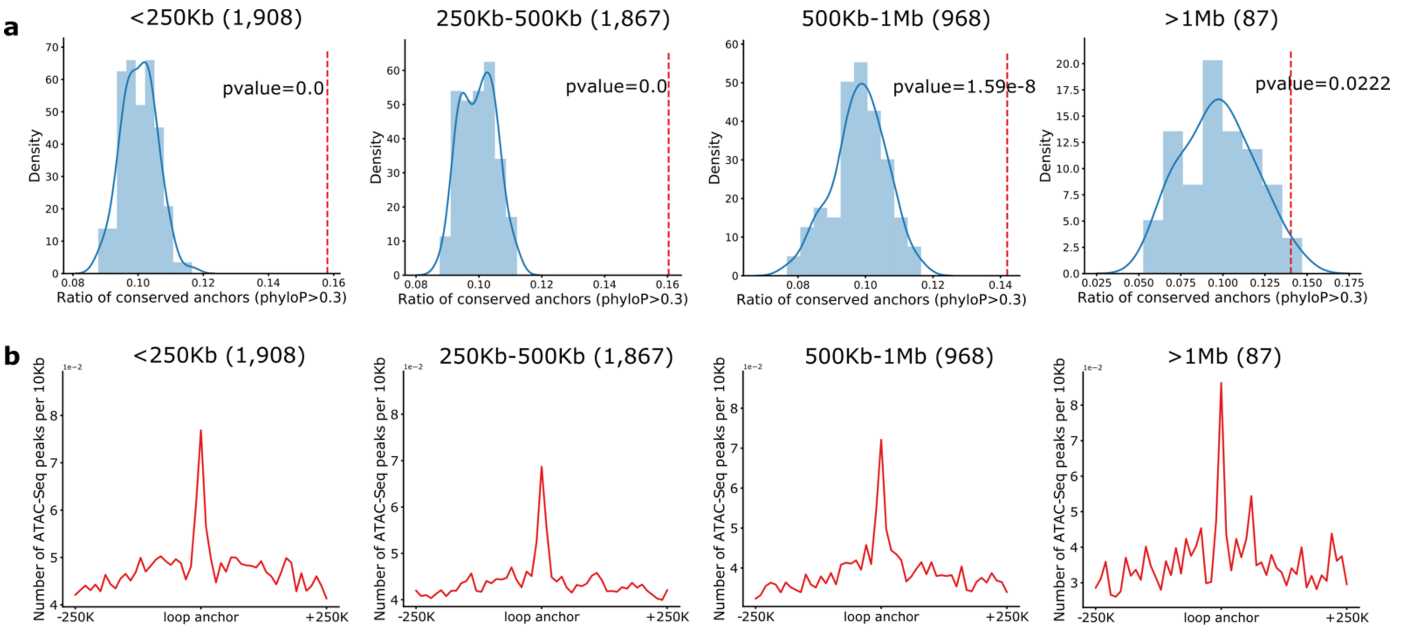

Supplementary Figure 9 | Peakachu-specific loops are evolutionarily conserved and enriched for ATAC-Seq signals. For this analysis, we used the loops predicted in GM12878 at 10kb resolution. **a**. We generated 100 sets of simulated loops matching the Peakachu-predicted loops. Each randomly generated set contains the same number of Peakachu-predicted loops and we calculated the ratio of loop anchors that are conserved in each set. As we performed the simulation 100 times, we plotted the density distribution of ratio of conserved anchors for each simulation (blue bar). The red vertical line is the conservation value for Peakachu-predicted loops. We observed that the loop anchors predicted by Peakachu are significantly more conserved than the random background. The four panels represent loops at each genomic distance. The number of loops is labeled within the parentheses in each case. And the p-values were calculated using one-sided Z-test. **b**. Chromatin accessibility around loop anchors (+/- 250Kb) at each genomic distance. We counted the average number of ATAC-Seq peaks for each 10Kb bin. Source data are available in the Source Data file.

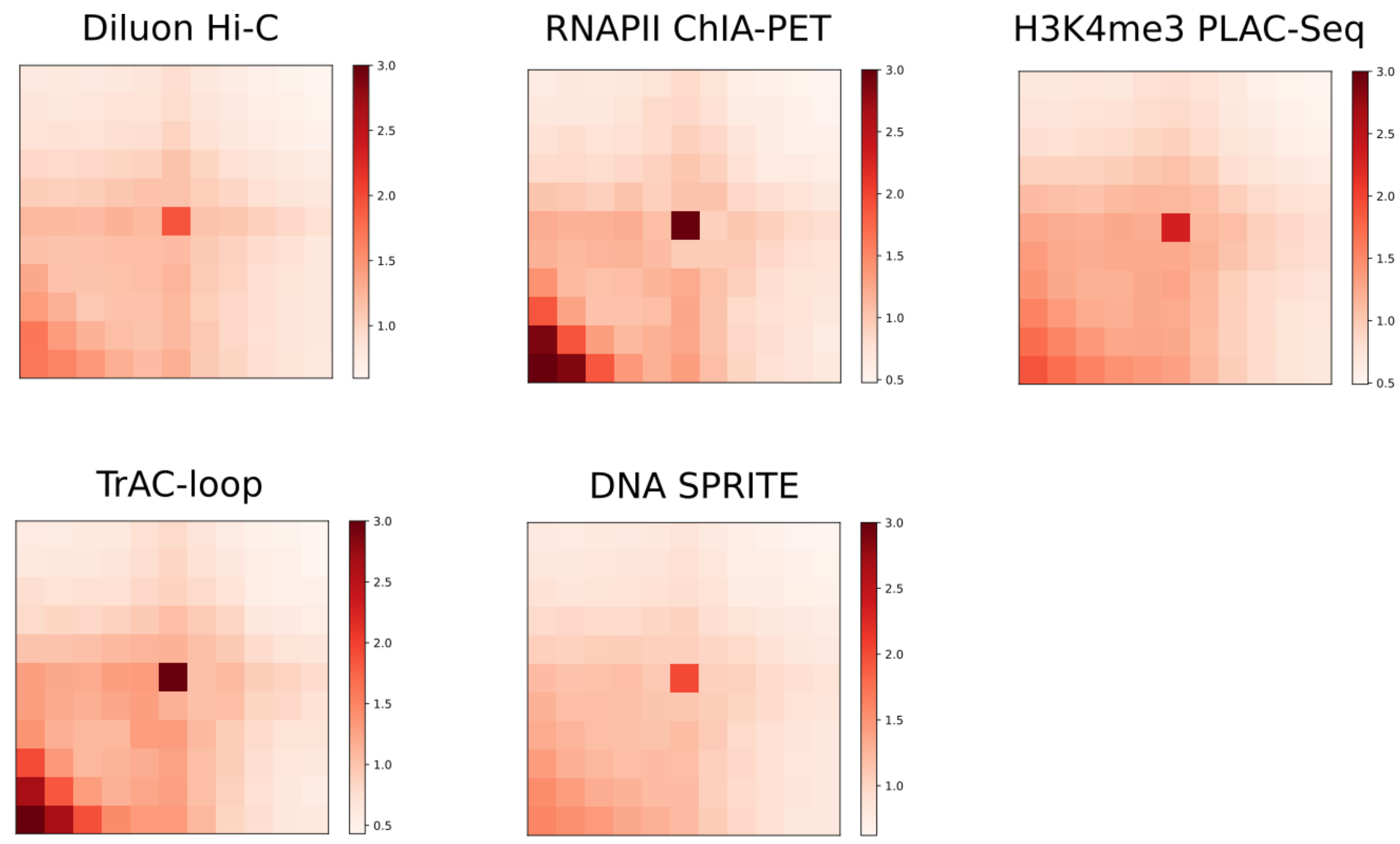

Supplementary Figure 10 | Peakachu-specific loops in GM12878 (compared with HiCCUPS and Fit-Hi-C) have support from five other orthogonal platforms in the same cell type. Source data are available in the Source Data file.

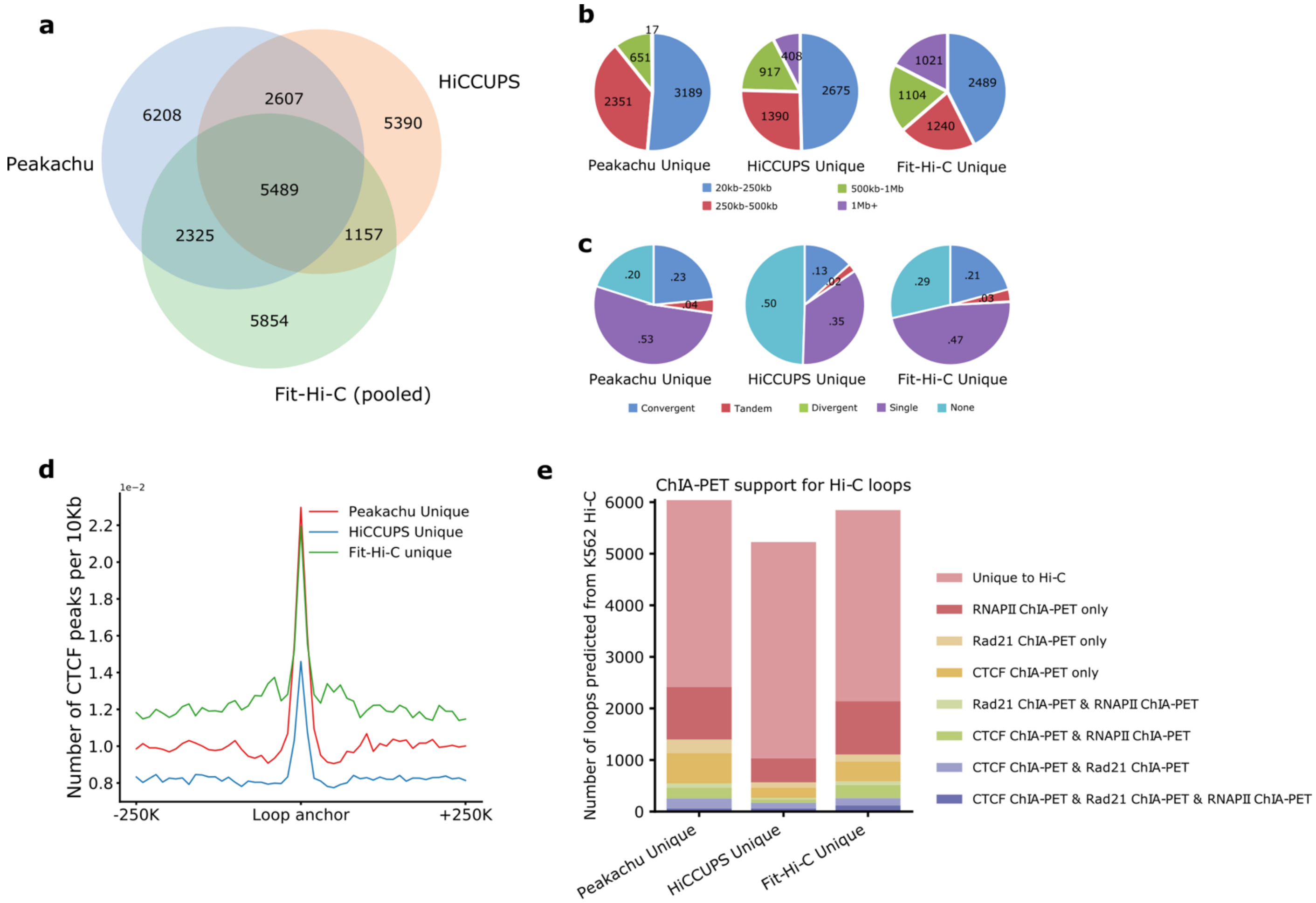

Supplementary Figure 11 | Comparisons of Peakachu, HiCCUPS and Fit-Hi-C in K562 Hi-C. a. Venn diagram of loops detected by different methods. **b-d.** Distance distributions and CTCF binding patterns of loops uniquely detected by each method. **e**. Orthogonal dataset support of unique loops detected by each method. Source data are available in the Source Data file.

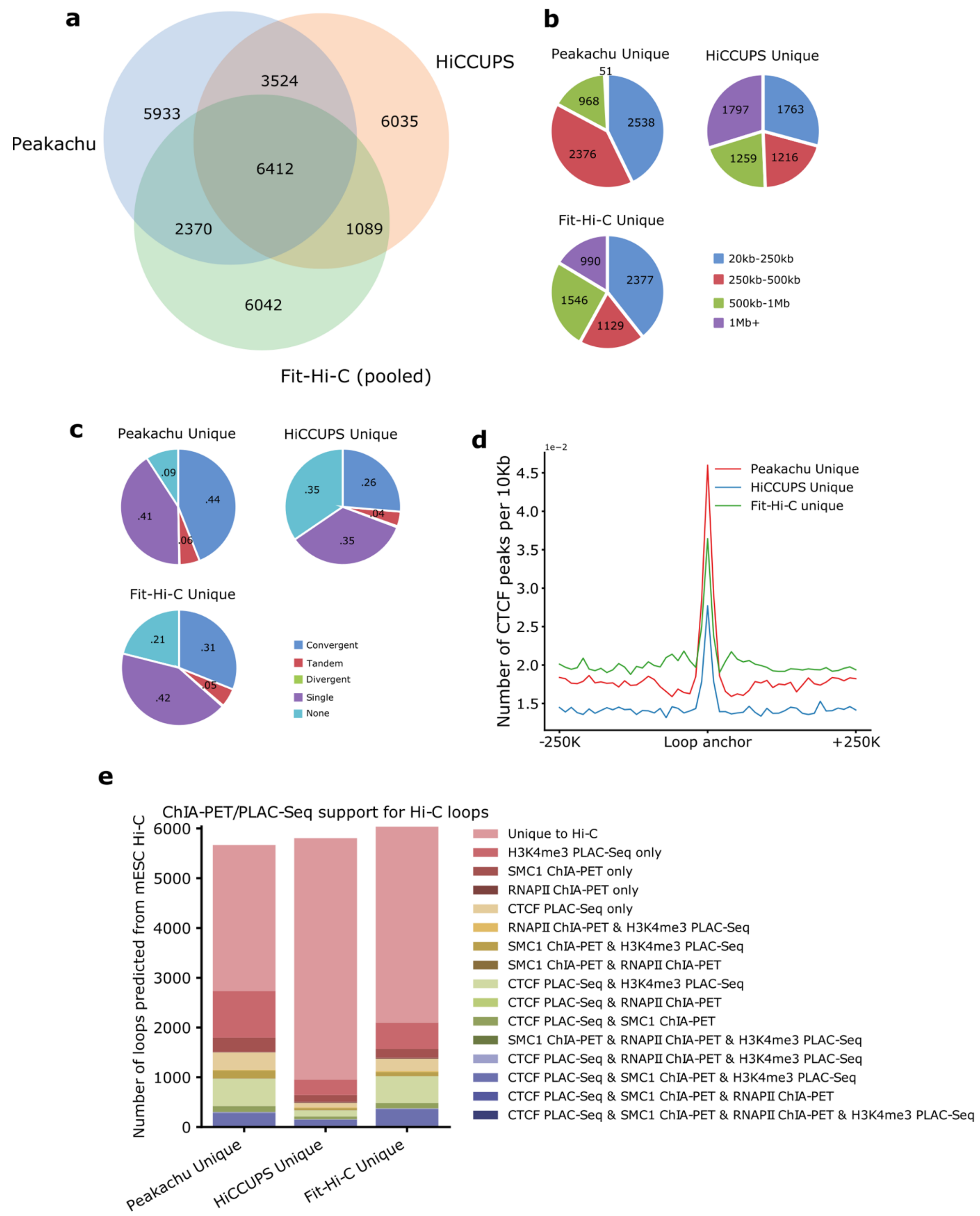

Supplementary Figure 12 | Comparisons of Peakachu, HiCCUPS and Fit-Hi-C in mESC Hi-C. a. Venn diagram of loops detected by different methods. **b-d.** Distance distributions and CTCF binding patterns of loops uniquely detected by each method. **e**. Overlap loops uniquely detected by each method with ChIA-PET/PLAC-Seq interactions. Source data are available in the Source Data file.

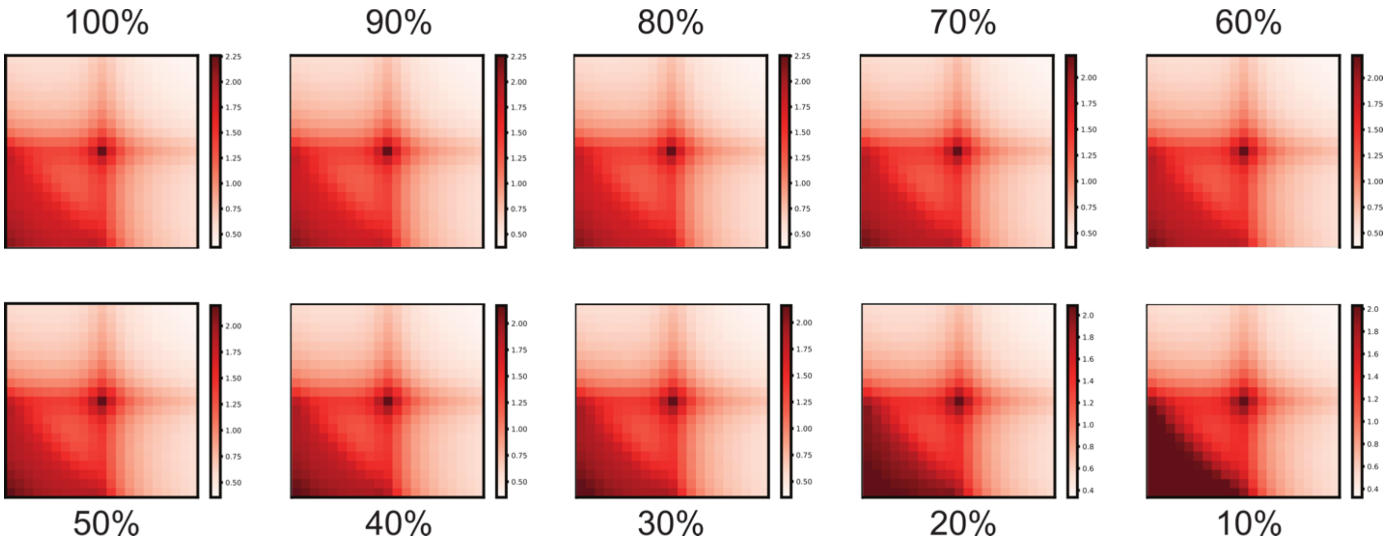

Supplementary Figure 13 | APA profiles of Peakachu loops predicted in down-sampled GM12878 Hi-C maps. Source data are available in the Source Data file.

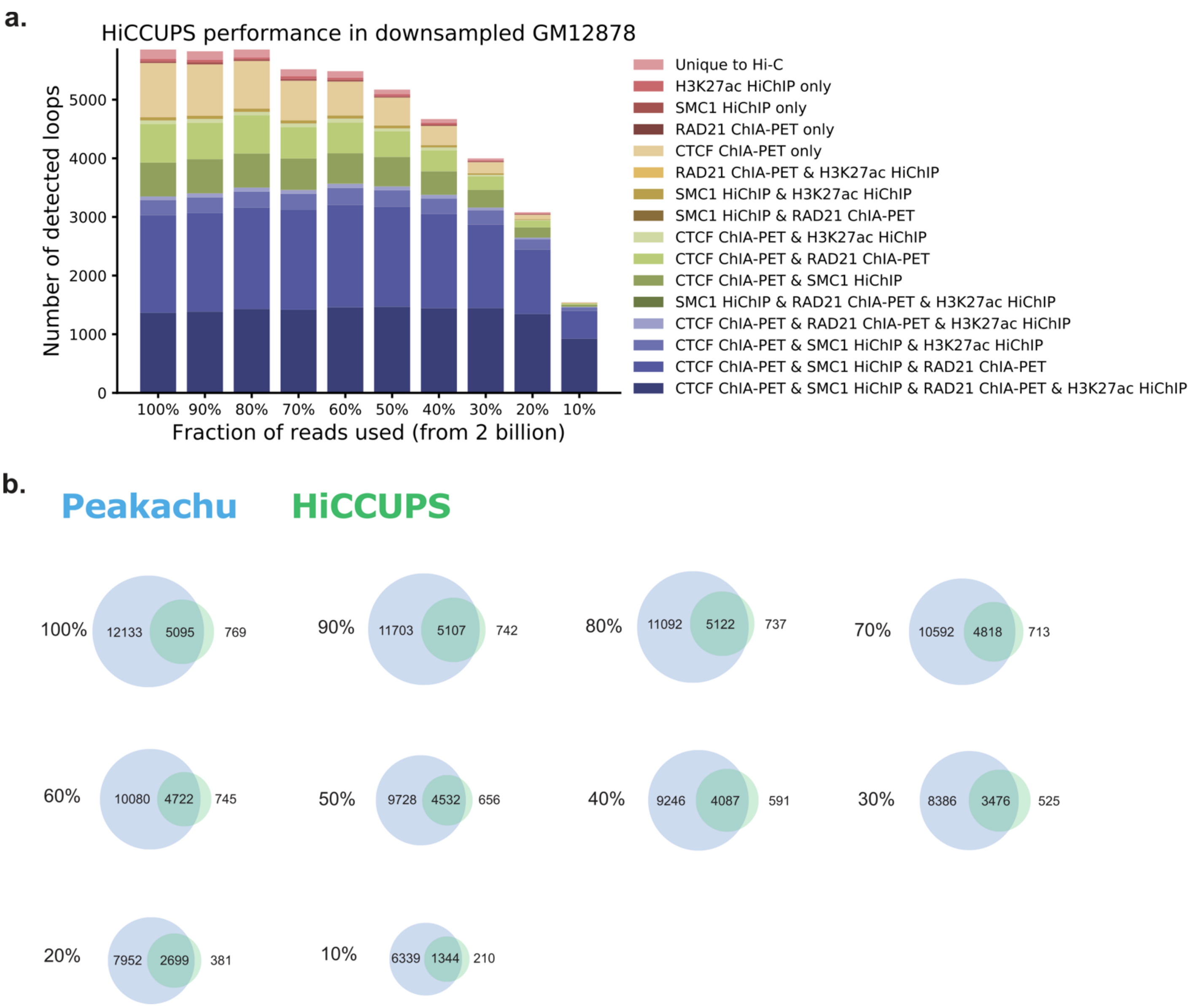

Supplementary Figure 14 | Performance of HiCCUPS and concordance with Peakachu for down-sampled GM12878 contact maps. HiCCUPS was run with default parameters. **a.** Overlap of HiCCUPS interactions with ChIA-PET and HiChIP datasets for predictions in GM12878 at different read depths**. b.** Overlap with Peakachu loops predicted from the same contact maps are displayed as Venn diagrams. Source data are available in the Source Data file.

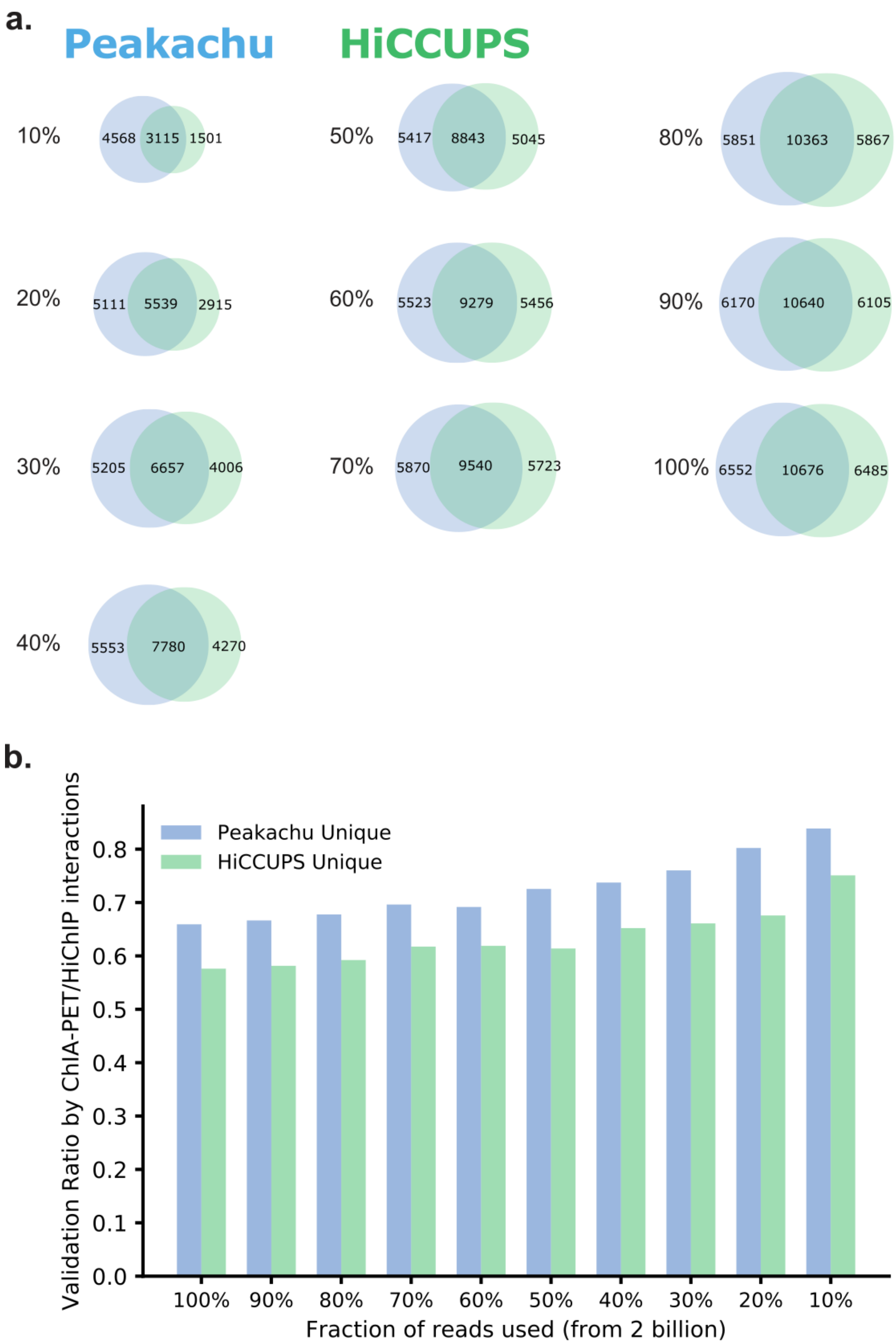

Supplementary Figure 15 | Comparison of HiCCUPS and Peakachu at different sequencing depths. We adjusted the HiCCUPS parameters so that it produced similar number of loops as Peakachu. **a**. Venn diagrams comparing Peakachu and HiCCUPS loops. **b**. Ratio of Peakachu-specific and HiCCUPS-specific loops that can be validated by at least one orthogonal dataset. Source data are available in the Source Data file.

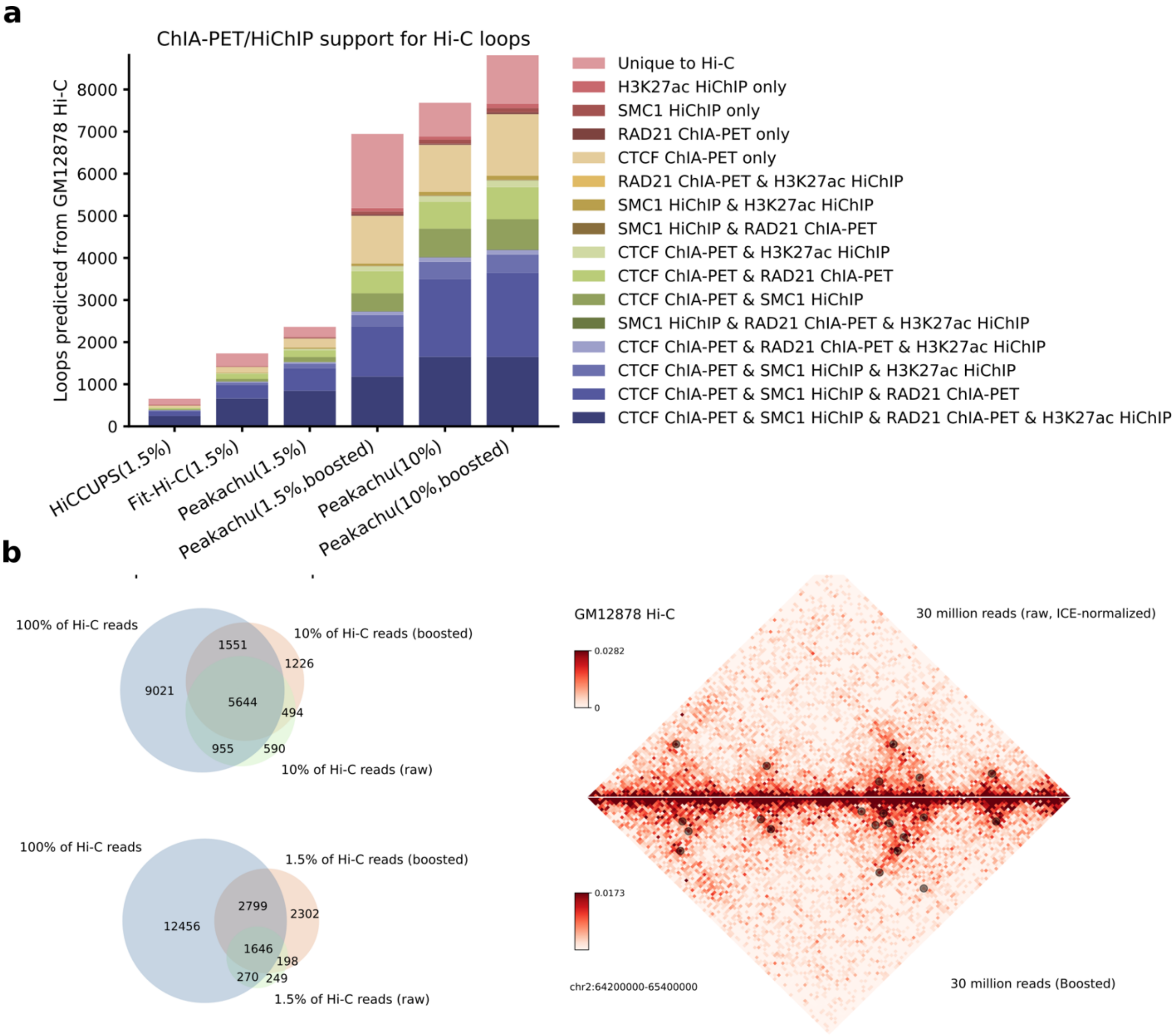

Supplementary Figure 16 | Performance of HiCCUPS, Fit-Hi-C, and Peakachu on augmented Hi-C data matrix by Boost-HiC. We first down-sampled the Hi-C in GM12878 to 1.5% and 10%, and then enhanced them with Boost-HiC. **a**. Validation rate of predicted loops by orthogonal ChIA-PET/HiChIP datasets. The first three columns are the prediction by directly applying HiCCUPS, Fit-Hi-C, and Peakachu. For the fourth and sixth columns (Peakachu 1.5% boosted, 10% boosted), we first enhanced the down-sampled Hi-C matrix with Boost-HiC, and trained the model in the boosted Hi-C matrix, and then made the prediction. **b**. Venn diagrams comparing Peakachu predictions using 10% (top) and 1.5% (bottom) of Hi-C reads with predictions using 100% of Hi-C reads. **c**. Visualization of loop predictions using 1.5% of Hi-C reads and boosted matrix. Source data are available in the Source Data file.

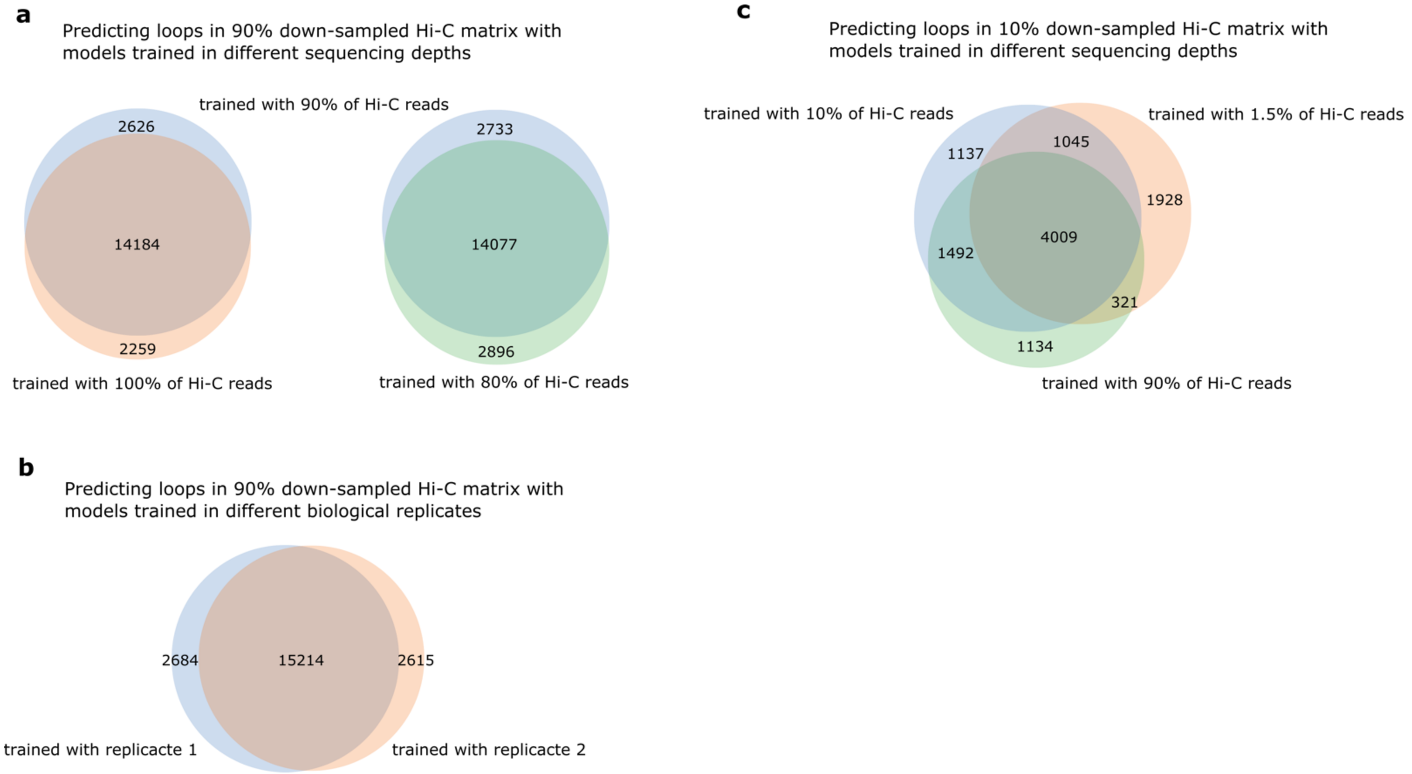

Supplementary Figure 17 | Evaluation of Peakachu model variations. a. Comparing predictions of models trained with 80% (1.6 billion), 90% (1.8 billion) and 100% (2 billion) reads on the matrix with 1.8 billion reads. **b.** Comparing predictions of models trained with two biological replicate Hi-C data (382 million vs 389 million) on the same matrix with 1.8 billion reads. **c.** Comparing predictions of models trained with 1.5% (30 million), 10% and 90% of Hi-C reads on the 10% down-sampled Hi-C matrix (200 million).

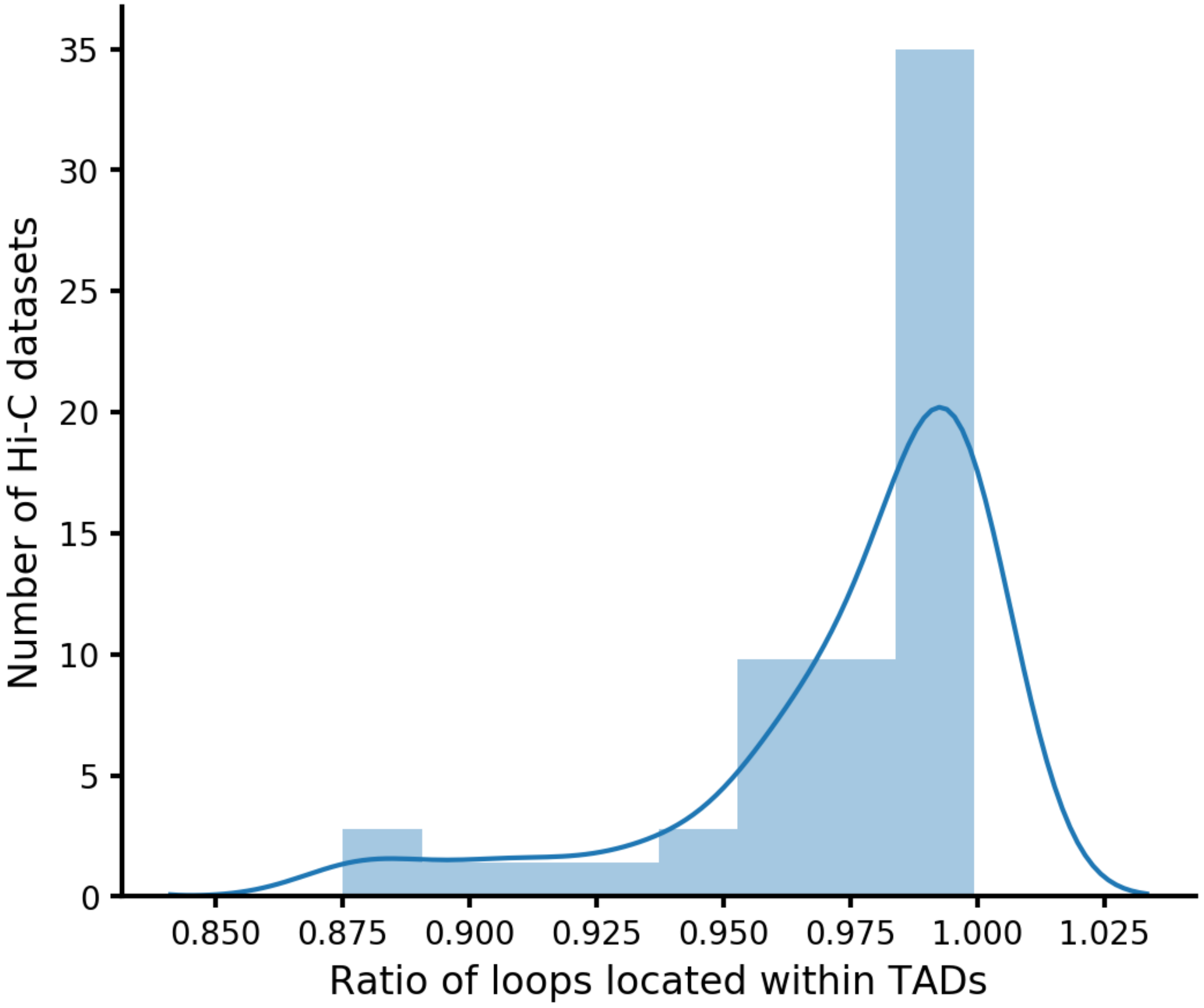

Supplementary Figure 18 | Ratio of Peakachu loops located within TADs for 56 Hi-C datasets. The TAD locations were downloaded from 3D genome browser (3dgenome.org).

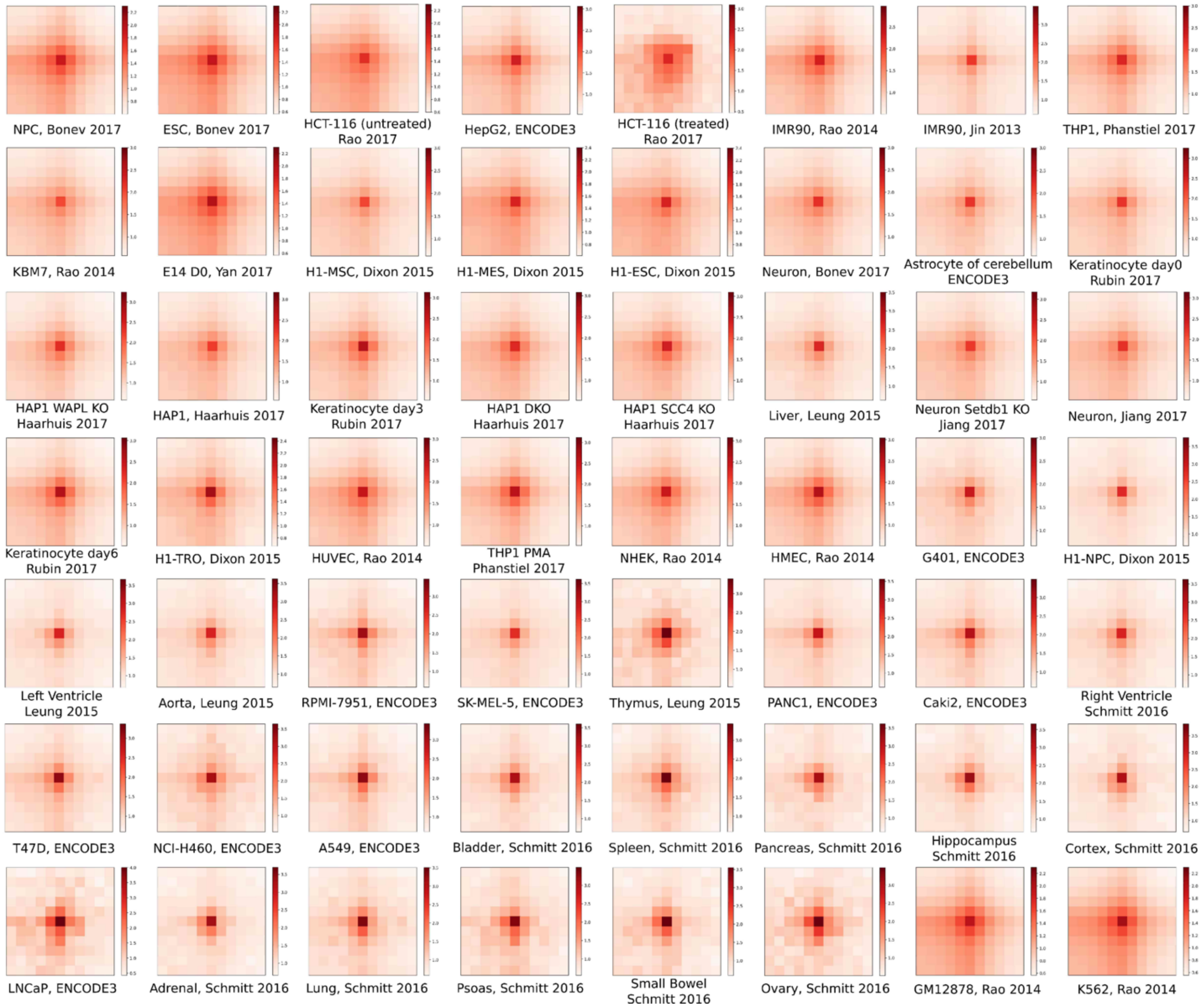

Supplementary Figure 19 | APA profiles of loops predicted in 56 Hi-C datasets.

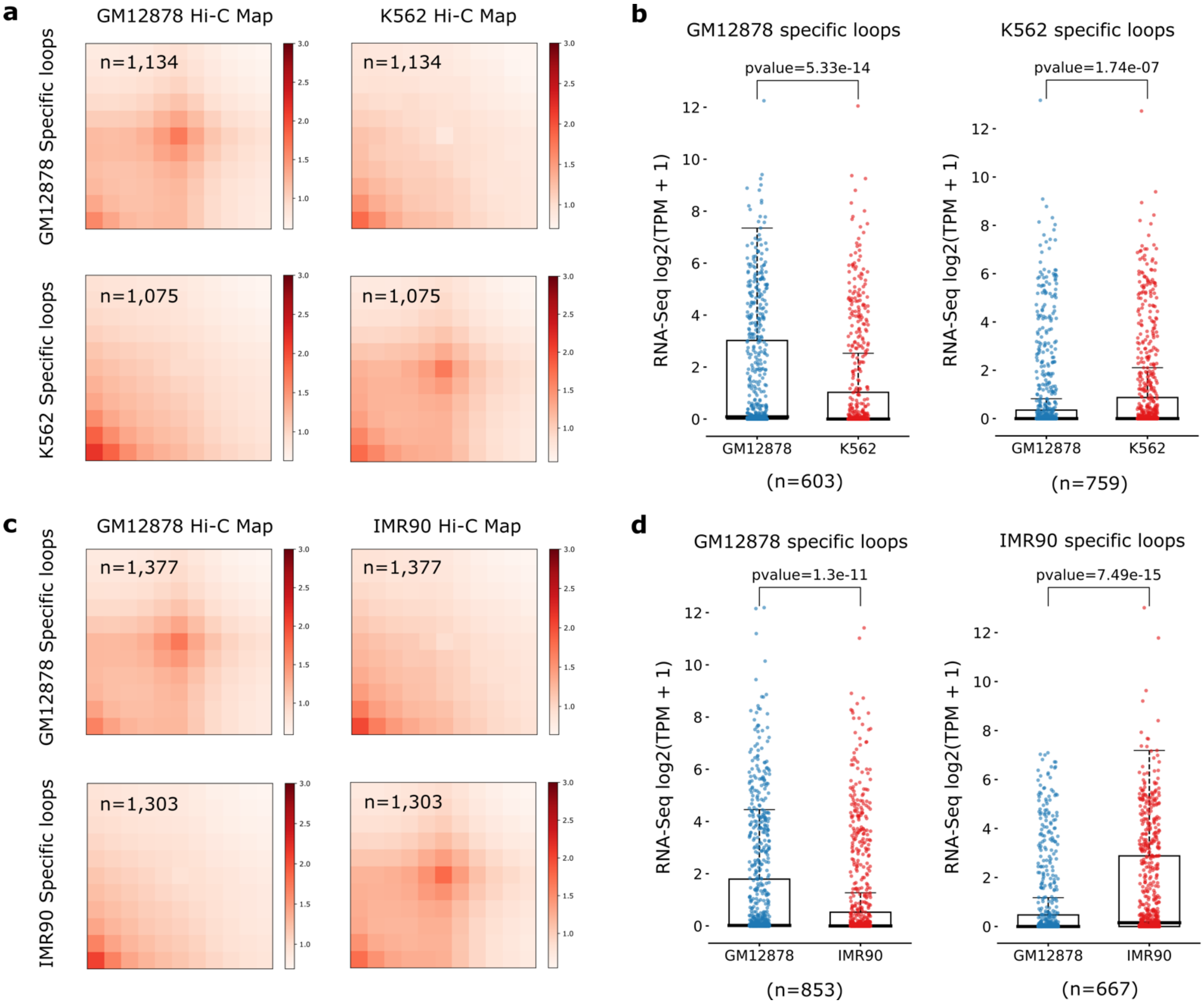

Supplementary Figure 20 | Differential Peakachu loops between cell lines and correlation with gene expressions. a. APA plots of GM12878 specific and K562 specific loops in either GM12878 Hi-C or K562 Hi-C map. **b**. Genes with promoter located within GM12878 specific loop anchors are specifically upregulated in GM12878 (left), while genes with promoter located within K562 specific loop anchors are specifically activated in K562. The quantile normalized RNA-Seq signals (Transcripts Per Kilobase Million, TPM) are presented in box-and-whisker plots for each comparison, where the box represents the interquartile range (IQR, Q3-Q1), the horizontal thick line represents the median, and the upper whisker extend to the last datum less than Q3+1.5×IQR. Each dot represents an individual gene. And the number of genes for each comparison is labeled at the bottom of each plot. The p-values were calculated using two-sided Wilcoxon signed-rank test. **c-d.** Similar analysis for differential loops between GM12878 and IMR90. Source data are available in the Source Data file.

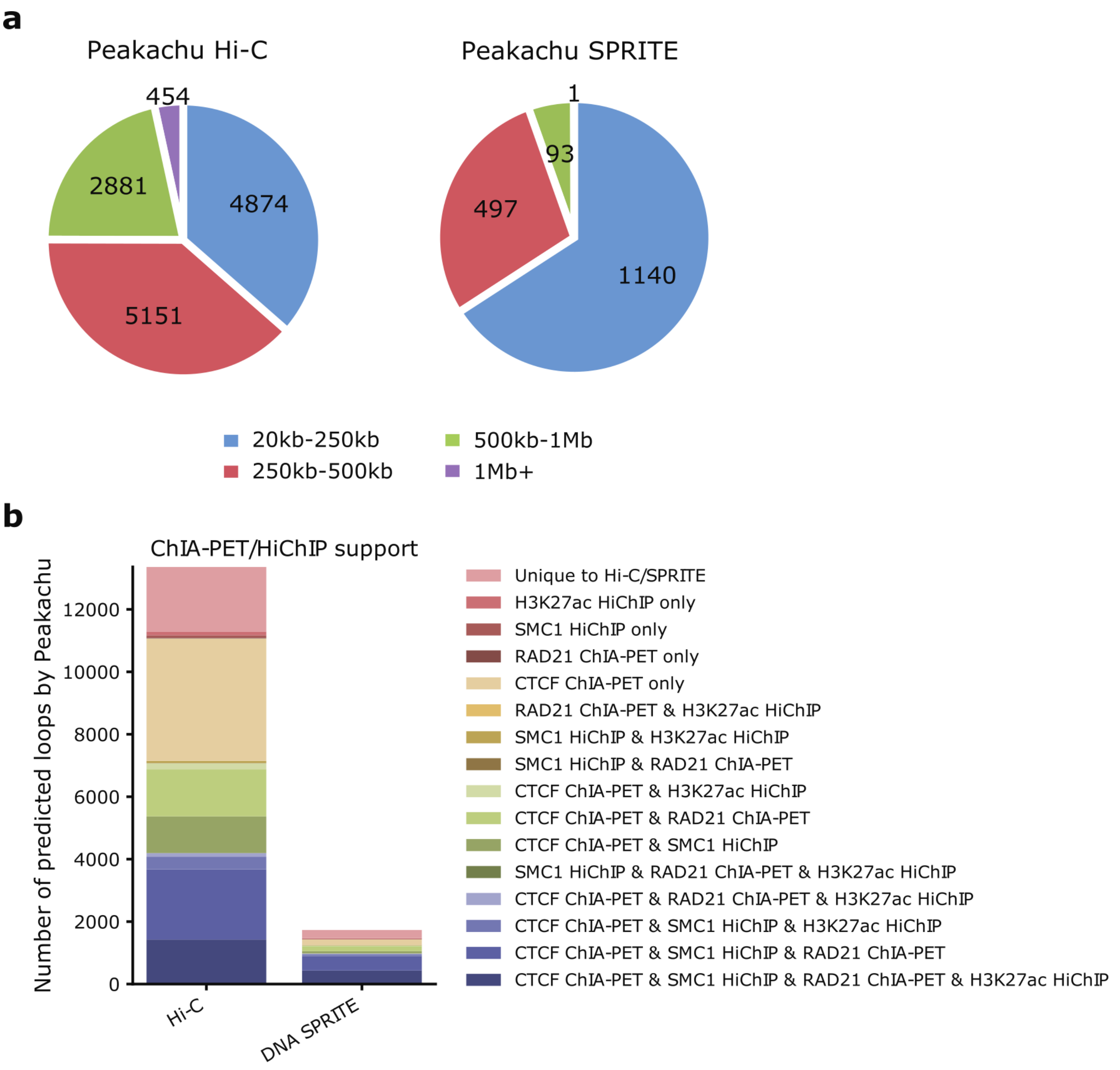

Supplementary Figure 21 | Distance distributions and validation rates for loops predicted in GM12878 Hi-C and DNA SPRITE datasets. Source data are available in the Source Data file.

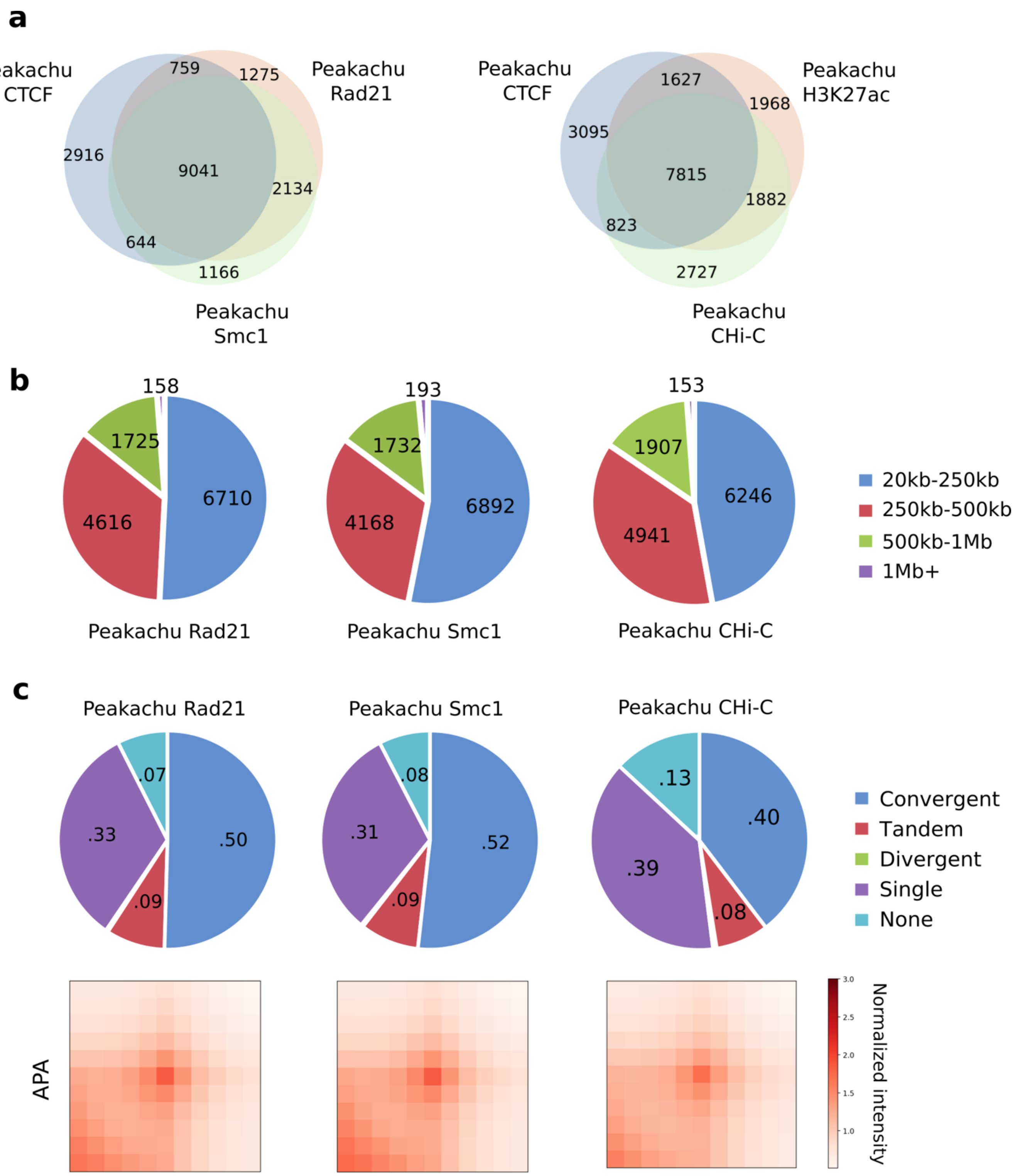

Supplementary Figure 22 | Comparisons of loops in GM12878 Hi-C predicted by models trained with different orthogonal datasets. **a**. Venn diagrams showing concordance of loop predictions by different Peakachu models. **b**. Distance distributions of loop predictions by models trained with Rad21 ChIA-PET, Smc1 HiChIP and promoter Capture Hi-C interactions. **c**. CTCF binding patterns and APA plots for loops predicted by different models. Source data are available in the Source Data file.

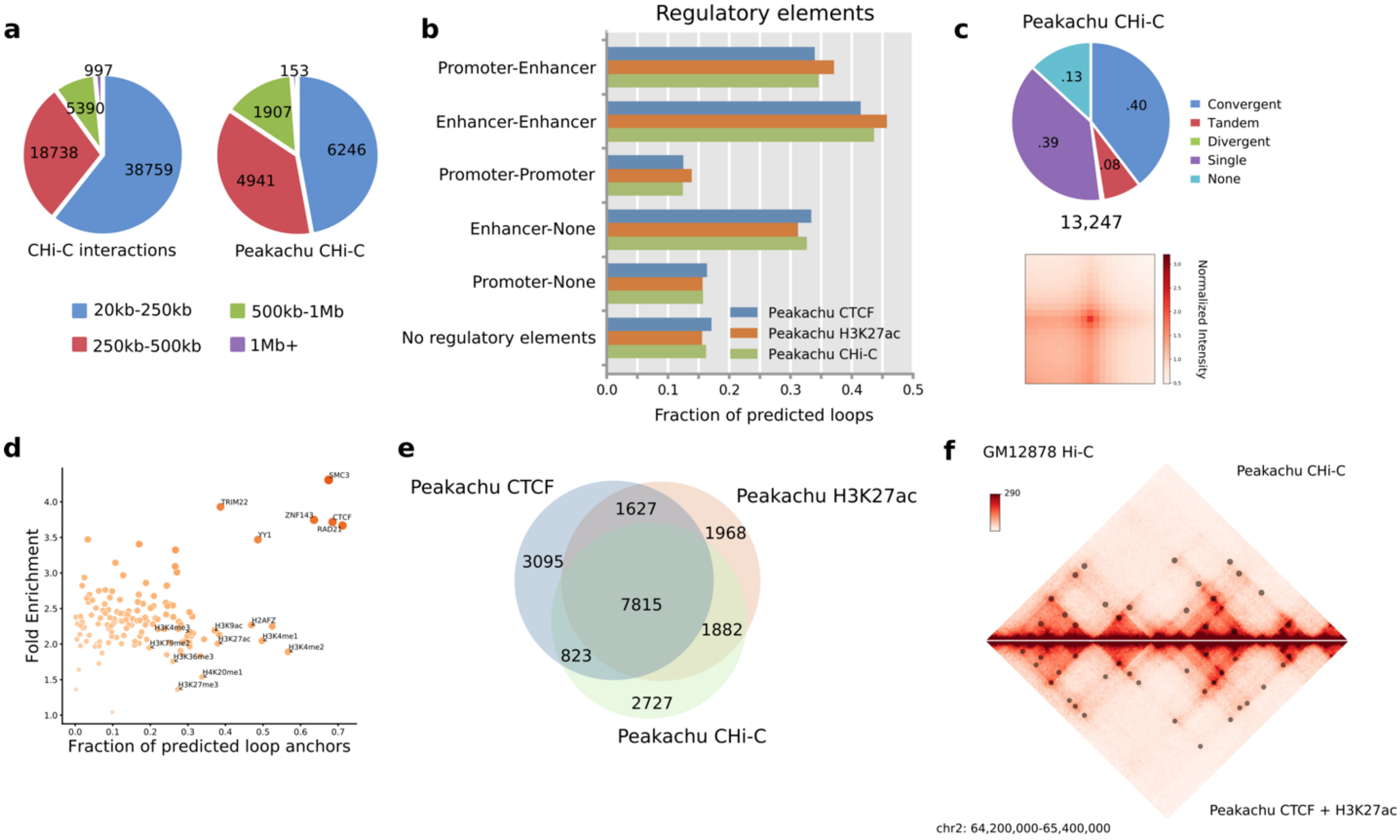

Supplementary Figure 23 | Loop predictions in GM12878 Hi-C using a model trained with promoter Capture Hi-C interactions. **a**. Distance distributions of the interactions used in training and the predicted loops. **b**. Proportion of predicted loops with different regulatory element combinations at anchor loci. **c**. CTCF motif orientation and APA plot of the predicted loops. **d**. Fraction of loop anchors bound versus fold enrichment for 133 transcription factors and 10 histone modifications. **e**. Overlap of loops predicted by Peakachu models trained with different orthogonal interactions. **f**. Visualization of predicted loops on the region (chr2: 64,200,000 – 65,400,000). Source data are available in the Source Data file.

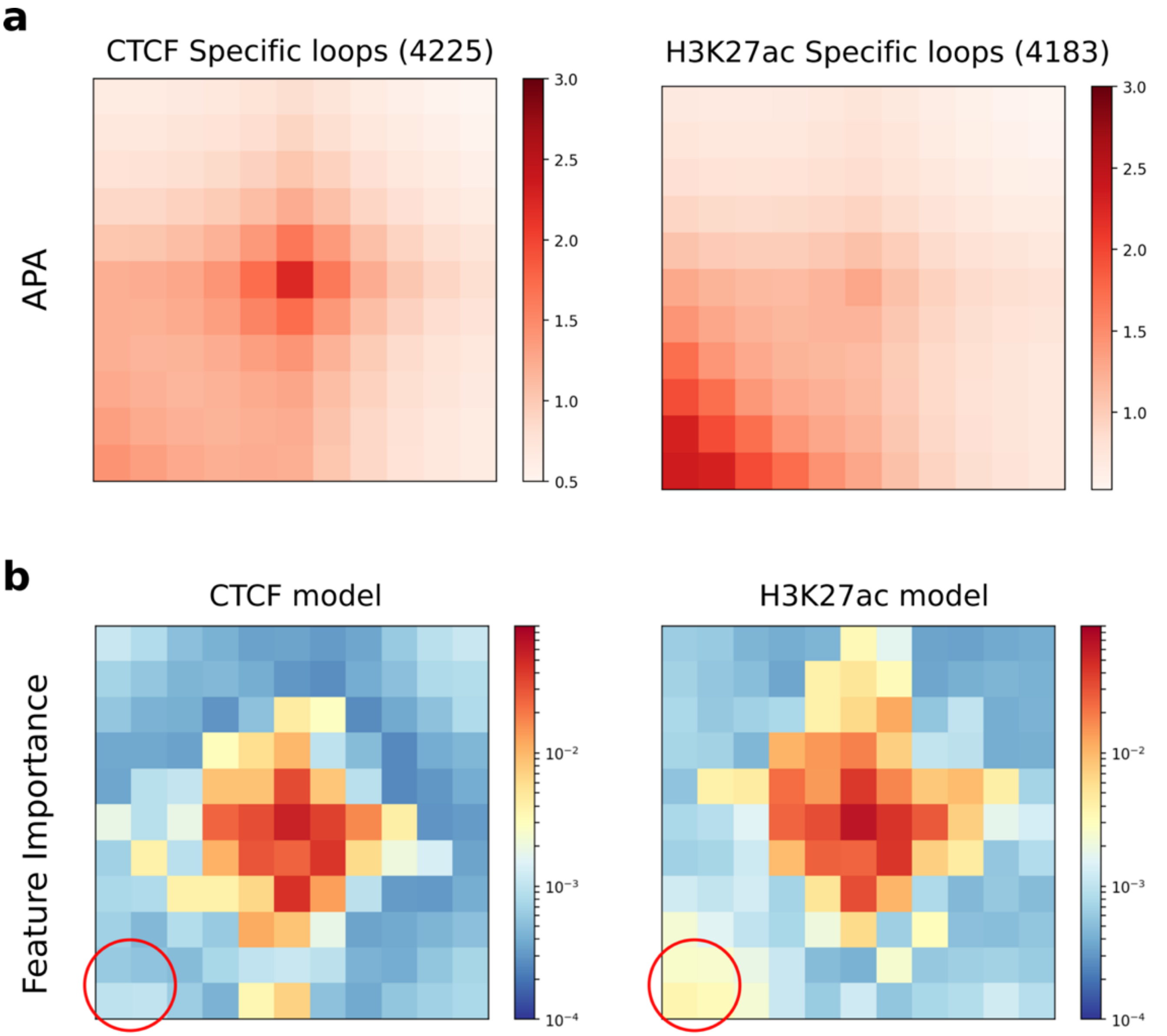

Supplementary Figure 24 | Different features drive the predictions for Peakachu CTCF and H3K27ac models. **a**. APA plots for loops uniquely predicted by CTCF or H3K27ac models in GM12878 Hi-C. The intensity of H3K27ac loops can be much lower than the lower-left region. **b**. The feature importance metric from random forests showing which pixels drive the classification most strongly. Source data are available in the Source Data file.

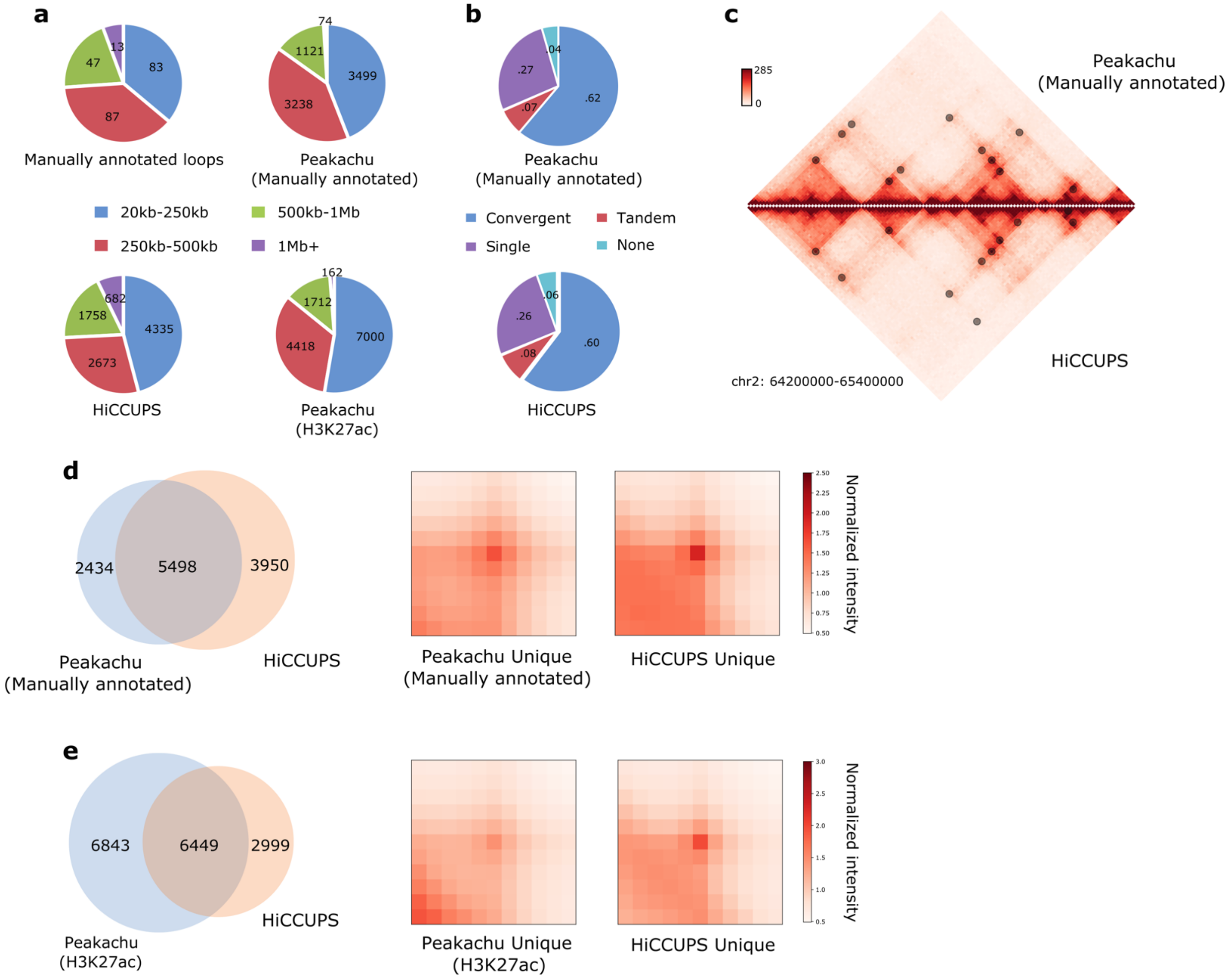

Supplementary Figure 25 | Loop predictions with the model trained with 230 manually annotated loops in GM12878 Hi-C. **a**. Distance distributions of loops predicted by manually selected model, HiCCUPS and Peakachu. **b**. CTCF binding patterns of Peakachu and HiCCUPS loops. **c**. Visualization of Peakachu and HiCCUPS loops within a random region. **d**. Comparison of Peakachu (trained with manually annotated loops) loops and HiCCUPS. APA plots were calculated for loops uniquely predicted by Peakachu or HiCCUPS. **e**. Comparison of Peakachu (trained with H3K27ac interactions) loops with HiCCUPS. Source data are available in the Source Data file.

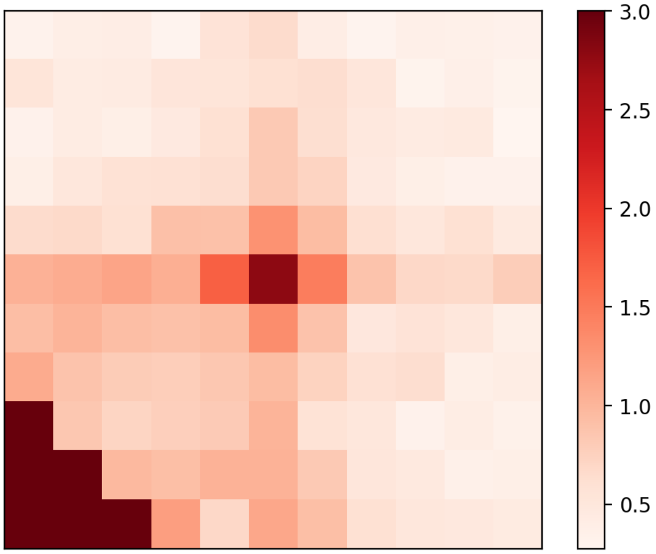

Supplementary Figure 26 | APA plot showing Peakachu predictions trained by CTCF and H3K27ac are enriched for YY1-mediated loops. Here, we plotted the YY1 ChIA-PET data in mESC as 10Kb heatmap for 18,239 Peakachu-predicted loops in the same cell line.

a

Machine learning frameworks	MCC	ACC	AUC	Training time (hr:min:sec)
Gaussian Naïve	0.518	0.757	0.869	00:00:11
Perceptron	0.709	0.851	0.939	00:09:37
Logistic Regression	0.829	0.914	0.969	09:54:37
SVM (linear)	0.840	0.920	0.973	01:22:25
SVM (rbf)	-	-	-	>48h (terminated)
Random Forest	0.867	0.934	0.981	02:15:32

1. # of positive training set: 54,834; # of negative set: 58,664
2. MCC: Matthews correlation coefficient
3. ACC: Accuracy
4. AUC: Area Under the ROC Curve

b

Machine learning frameworks	MCC	ACC	AUC	Training time (hr:min:sec)
Gaussian Naïve	0.634	0.817	0.890	00:00:01
Perceptron	0.764	0.882	0.948	00:00:24
Logistic Regression	0.839	0.919	0.970	00:38:42
SVM (linear)	0.865	0.932	0.975	00:07:51
SVM (rbf)	0.870	0.935	0.976	01:13:19
Random Forest	0.870	0.935	0.980	00:08:27

1. # of positive training set: 4,875; # of negative set: 5,148
2. MCC: Matthews correlation coefficient
3. ACC: Accuracy
4. AUC: Area Under the ROC Curve

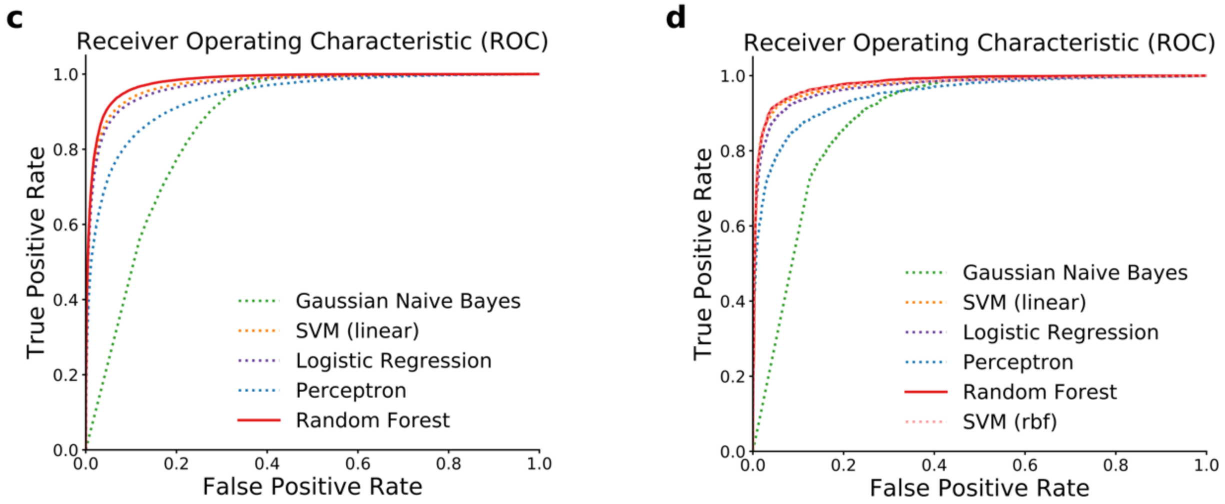

Supplementary Figure 27 | Performance of different machine learning frameworks in predicting loops. Models were trained with either CTCF ChIA-PET (**a, c**) or H3K27ac HiChIP interactions (**b, d**). Predictions for each chromosome used the model trained with data from the rest of the 22 chromosomes (cross-validation). Various measurements were used to evaluate the performance of a framework, including the elapsed time for training, Matthews correlation coefficient (MCC), prediction accuracy (ACC), receiver operating characteristic (ROC) curve and the area under the ROC curve (AUC).
