# Supplementary material for: A supervised learning framework for chromatin loop detection in genome-wide contact maps
Source: Nat Commun. 2020 Jul 9;11:3428. doi: 10.1038/s41467-020-17239-9 (PMC7347923; doi:10.1038/s41467-020-17239-9)
Supplement: Supplementary file 2 — Description of Additional Supplementary Files [file 41467_2020_17239_MOESM2_ESM.pdf]

Description of Additional Supplementary Files

File Name: Supplementary Data 1
Description: Collected datasets for Peakachu model training and validation.

File Name: Supplementary Data 2
Description: Detailed information about the predicted loops in 56 Hi-C datasets.

File Name: Supplementary Data 3
Description: Coordinates of 230 manually selected loops in GM12878 Hi-C map.
