# Supplementary material for: A supervised learning framework for chromatin loop detection in genome-wide contact maps
Source: Nat Commun. 2020 Jul 9;11:3428. doi: 10.1038/s41467-020-17239-9 (PMC7347923; doi:10.1038/s41467-020-17239-9)
Supplement: Supplementary file 6 — Reporting Summary [file 41467_2020_17239_MOESM6_ESM.pdf]

nature research

Corresponding author(s): Feng Yue

Last updated by author(s): Jun 3, 2020

Reporting Summary

Nature Research wishes to improve the reproducibility of the work that we publish. This form provides structure for consistency and transparency in reporting. For further information on Nature Research policies, see our Editorial Policies and the Editorial Policy Checklist.

Statistics

For all statistical analyses, confirm that the following items are present in the figure legend, table legend, main text, or Methods section.

n/a	Confirmed	
☒	☐	The exact sample size (*n*) for each experimental group/condition, given as a discrete number and unit of measurement
☒	☐	A statement on whether measurements were taken from distinct samples or whether the same sample was measured repeatedly
☐	☒	The statistical test(s) used AND whether they are one- or two-sided<br>*Only common tests should be described solely by name; describe more complex techniques in the Methods section.*
☒	☐	A description of all covariates tested
☒	☐	A description of any assumptions or corrections, such as tests of normality and adjustment for multiple comparisons
☒	☐	A full description of the statistical parameters including central tendency (e.g. means) or other basic estimates (e.g. regression coefficient) AND variation (e.g. standard deviation) or associated estimates of uncertainty (e.g. confidence intervals)
☐	☒	For null hypothesis testing, the test statistic (e.g. *F*, *t*, *r*) with confidence intervals, effect sizes, degrees of freedom and *P* value noted<br>*Give P values as exact values whenever suitable.*
☒	☐	For Bayesian analysis, information on the choice of priors and Markov chain Monte Carlo settings
☒	☐	For hierarchical and complex designs, identification of the appropriate level for tests and full reporting of outcomes
☒	☐	Estimates of effect sizes (e.g. Cohen's *d*, Pearson's *r*), indicating how they were calculated

Our web collection on statistics for biologists contains articles on many of the points above.

Software and code

Policy information about availability of computer code

Data collection	We used a custom script to batch download the ChIP-Seq peaks for 133 TFs and 10 histone modifications. The code and the metadata are available at https://github.com/tariks/peakachu/tree/master/analysis/ENCODE. For other data, we downloaded individually according the data sources summarized in Supplementary Data 1.
Data analysis	Software versions: cooler 0.8.5; bedtools v2.28.0; Juicer 1.11.09 for HiCCUPS loop detections; Fit-Hi-C 2.0.7; deeptools 3.1.3; hicpeaks 0.3.4 for generating APA plots. The peakachu source code, the down-sampling script, the interaction pooling scripts and the pre-trained models are available in GitHub under the MIT License: https://github.com/tariks/peakachu.

For manuscripts utilizing custom algorithms or software that are central to the research but not yet described in published literature, software must be made available to editors and reviewers. We strongly encourage code deposition in a community repository (e.g. GitHub). See the Nature Research guidelines for submitting code & software for further information.

Data

Policy information about availability of data

All manuscripts must include a data availability statement. This statement should provide the following information, where applicable:

- Accession codes, unique identifiers, or web links for publicly available datasets
- A list of figures that have associated raw data
- A description of any restrictions on data availability

All datasets used in this work are summarized in Supplementary Data 1. The Hi-C contact maps of GM12878 and K562 were obtained from ftp://cooler.csail.mit.edu/coolers/hg19/. The DNA SPRITE contact map for GM12878 was obtained from 4DN data portal with accession code 4DNFIUOOYQC3. The Hi-C contact map of H1-ESC was obtained from 4DN data portal with accession code 4DNFI6HDY7WZ. The Micro-C contact map of H1-ESC was obtained from 4DN data portal with accession code 4DNFI9GMP2J8. The CTCF ChIA-PET interactions in GM12878 were obtained from Tang et al.. The Rad21ChIA-PET interactions in

GM12878 were obtained from Heidari et al.. The SMC1 HiChIP interactions in GM12878 were obtained from Mumbach et al.. The H3K27ac HiChIP interactions in GM12878 were obtained from Mumbach et al.. The promoter Capture Hi-C interactions in GM12878 were obtained from Cairns et al.. The CTCF ChIA-PET interactions in K562 were obtained from ENCODE with accession code ENCFF001THV. The SMC1 HiChIP interactions in mouse ESC were obtained from Mumbach et al.. The CTCF ChIA-PET interactions in H1-ESC were obtained from 4DN data portal with accession code 4DNESR9S8R38. All aforementioned positive training datasets can be found at https://github.com/tariks/peakachu/tree/master/training-sets. The enhancer and promoter loci in GM12878, K562, H1-ESC and mouse ESC were extracted from public ChromHMM annotations in ENCODE and can be found at https://github.com/tariks/peakachu/tree/master/analysis/annotations. The genome-wide CTCF motifs in human and mouse were obtained from https://bcm.app.box.com/v/juicerawsmirror/folder/11363582187. The predicted chromatin loops in 56 Hi-C datasets can be downloaded from the 3D Genome Browser (http://3dgenome.org).

Field-specific reporting

Please select the one below that is the best fit for your research. If you are not sure, read the appropriate sections before making your selection.

☒ Life sciences ☐ Behavioural & social sciences ☐ Ecological, evolutionary & environmental sciences

For a reference copy of the document with all sections, see nature.com/documents/nr-reporting-summary-flat.pdf

Life sciences study design

All studies must disclose on these points even when the disclosure is negative.

Sample size	We do not need to determine the sample size in this study.
Data exclusions	No data were excluded from the analysis.
Replication	The models trained on different biological replicates are comparable. (Supplementary Figure 17b).
Randomization	This is not relevant to our study. Our study does not involve any allocations of samples/organisms/participants.
Blinding	This not relevant to our study. Our study does not involve any group allocations.

Reporting for specific materials, systems and methods

We require information from authors about some types of materials, experimental systems and methods used in many studies. Here, indicate whether each material, system or method listed is relevant to your study. If you are not sure if a list item applies to your research, read the appropriate section before selecting a response.

Materials & experimental systems

n/a	Involved in the study	
☒	☐	Antibodies
☒	☐	Eukaryotic cell lines
☒	☐	Palaeontology and archaeology
☒	☐	Animals and other organisms
☒	☐	Human research participants
☒	☐	Clinical data
☒	☐	Dual use research of concern

Methods

n/a	Involved in the study	
☒	☐	ChIP-seq
☒	☐	Flow cytometry
☒	☐	MRI-based neuroimaging
